# Design, synthesis and photophysical properties of novel star-shaped truxene-based heterocycles utilizing ring-closing metathesis, Clauson–Kaas, Van Leusen and Ullmann-type reactions as key tools

**DOI:** 10.3762/bjoc.17.96

**Published:** 2021-06-02

**Authors:** Shakeel Alvi, Rashid Ali

**Affiliations:** 1 Department of Chemistry, Jamia Millia Islamia, Jamia Nagar, Okhla, New Delhi-110025, India; Phone: +91-7011867613

**Keywords:** Clauson–Kaas reaction, heterocycles, ring-closing metathesis, truxene, Ullmann-type coupling, Van Leusen reaction

## Abstract

Design, synthesis and properties of polycyclic aromatic hydrocarbons (PAHs) has historically attracted a considerable interdisciplinary interest from both fundamental as well as applied viewpoint on account of their wonderful optoelectronic properties. The scientific interest in two-dimensional star-shaped PAHs particularly in truxene architectures arises because of their high thermal stability, exceptional solubility and ease with which they can be constructed and modified. Therefore, bearing in mind a wide range of applications of truxene and its congeners, herein we reveal three novel distinctly different routes for the generation of *C*_3_-symmetric pyrrole-based truxene architectures by means of cyclotrimerization, ring-closing metathesis (RCM), Clauson–Kaas and Ullmann-type coupling reactions as key steps. Moreover, we have also assembled some other interesting heterocyclic systems possessing oxazole, imidazole, benzimidazole, and benzoxazole in the framework of truxene. Additionally, the preliminary photophysical properties (absorption and emission) for these versatile systems has been revealed.

## Introduction

The two-dimensional phenylene-based π-conjugated star-shaped architectures has recently attracted a tremendous attention of the researchers across the globe because of their inimitable physiochemical properties, processability and availability [1–4]. Interestingly, compared to the π-conjugated one-dimensional rod-shaped architectures, two-dimensional star-shaped systems hold, superior solubility, improve morphological, optical, electrical and film-forming properties because of the involvement of extra dimensionality. Amongst them, the heptacyclic truxene scaffold possessing three overlapping fluorene units and its congeners are of archetypal interest due to their potential applications ranging from organic thin‐film transistors (OTFTs), organic light-emitting diodes (OLEDs), dye‐sensitized solar cells (DSSCs), fluorescent probes, organic photovoltaics (OPVs) to the high hole mobility semiconductors, blue light-emitting materials, nonlinear optical materials and so forth [5–7]. More interestingly, the high fluorescence quantum yield as well as the excellent photoluminescence quantum efficiency in addition to extraordinary thermal stability are the most significant characteristics of these systems.

Interestingly, this versatile *C*_3_-symmetric aromatic framework can also formally be considered as a 1,3,5‐triphenylbenzene derivative enjoying with three methylene units clipped in such a manner that all the four benzene rings are in conjugation with coplanar arrangement, resulting strong π–π stacking in addition to the strong electron‐donating ability which in turns provide a hint for its utility as novel building block for the development of a range of advanced functional materials for innumerable applications [8–16]. Moreover, truxene has also been vividly used for the construction of fullerenes and their bowl-shaped fragments besides their applicability in the synthesis of dendrimers as well as *C*_3_-tripods and also in the host–guest chemistry [17–21]. This architecturally simple yet effective rigid system was first prepared in 1894 by Kipping [22] involving the trimerization of 3‐phenylpropionic acid under acidic conditions but surprisingly its futuristic applications in a myriad of areas boomed in recent past only. It is really amazing that how time has passed and a plethora of novel synthetic breakthroughs, coupled with a range of revolutionary applications has drastically transformed this realm of modern science and technology. Not surprisingly, till date limitless functionalized truxenes and their isomeric isotruxene derivatives have been reported in the literature [23–27]. Since, the chemistry of truxenes is undoubtedly of great interest, therefore there is always a pressing need to develop simple yet powerful synthetic strategies and also novel truxene-based functional materials with unique properties. Bearing in mind the importance of the truxene framework, herein, we report diverse *C*_3_-symmetric heterocyclic systems possessing the truxene scaffold in their structures by means of cyclotrimerization, ring-closing metathesis, Ullmann-type coupling, Clauson–Kaas, and Van Leusen reactions as crucial transformations.

## Synthetic Strategy

**Scheme 1:**
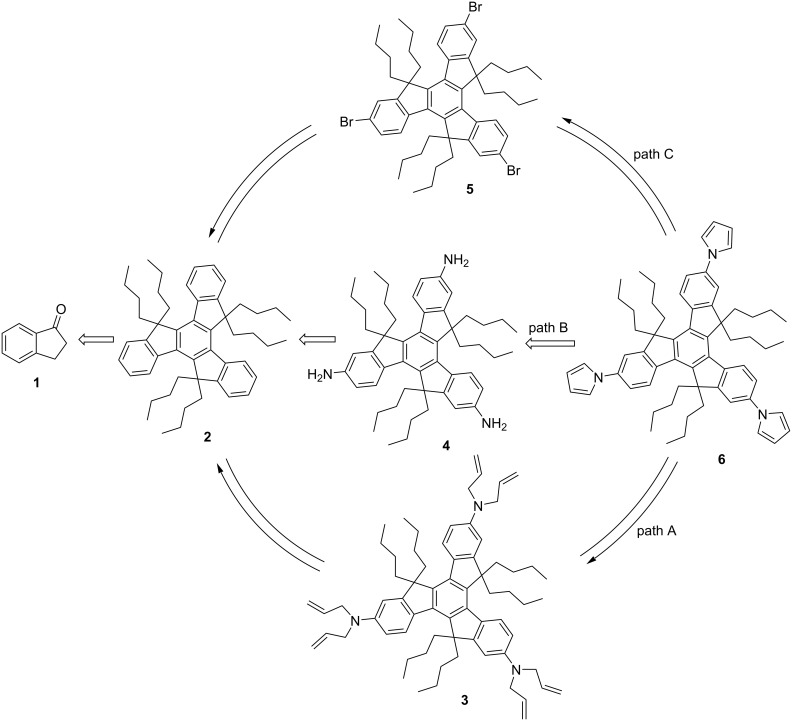
Retrosynthetic pathways to the pyrrole-based *C*_3_-symmetric truxene derivative **6**.

Basically, our endeavor toward the synthesis of truxene-based systems has been rooted in the development of new synthetic methodologies to assemble diverse polycyclic as well as spirocyclic structures. The present strategy to construct the pyrrole-based star-shaped truxene derivative **6** involve the three different retrosynthetic pathways A, B and C (Scheme 1). As can be seen from an inspection of Scheme 1, path A involves the transformation of the *N*-hexa-allylated system **3** to the required *C*_3_-symmetric tripyrroltruxene **6** by means of RCM in the presence of Grubbs′ first generation catalyst (G-I, **9**) followed by self-aromatization without the involvement of any oxidizing agent. Compound **3** was produced from the hexabutylated truxene system **2** involving nitration, reduction and *N*-allylation sequences, which in turn was assembled from the commercially available 1-indanone (**1**) utilizing cyclotrimerization and subsequent alkylation reactions. On the other hand, the same compound **6** was generated form triaminotruxene **4** using three-fold Clauson–Kaas reaction which in turn was assembled from the same starting material **1** by employing the acid-catalyzed cyclotrimerization, alkylation and subsequent reduction reaction sequences (path B, Scheme 1). In sharp contrast, as can be inspected from path C (Scheme 1) title compound **6** was also synthesized from the tribromotruxene derivative **5** by virtue of the Ullmann-type coupling reaction. The tribromotruxene derivative **5** was prepared from the same starting point **1** in three steps (cyclotrimerization, bromination and alkylation) using the literature reported procedures (Scheme 1). Hopefully, these three distinctly different crucial strategies could open-up the challenging opportunities to magnify the chemical space not only for the pyrrole derivatives but also of the truxene-based architectures, and the present contribution would definitely add value to both heterocyclic systems as well as to the truxene chemistry.

## Results and Discussion

**Scheme 2:**
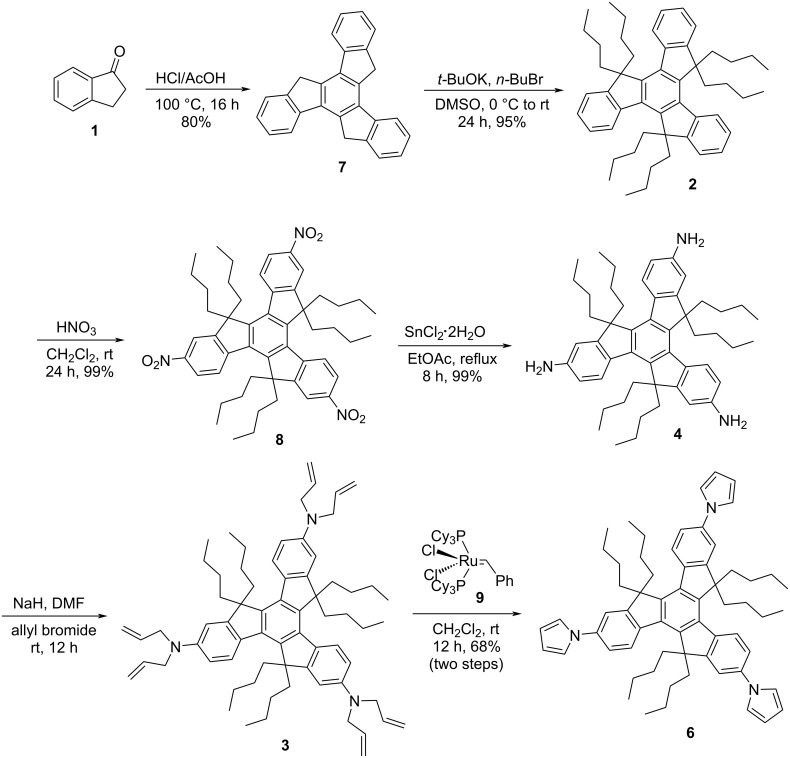
Synthesis of tripyrrolotruxene **6** via cyclotrimerization and RCM as crucial steps.

**Scheme 3:**
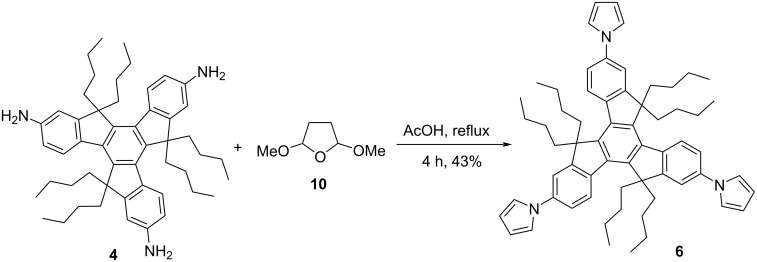
Synthesis of star-shaped molecule **6** utilizing the Clauson–Kaas pyrrole strategy.

**Scheme 4:**
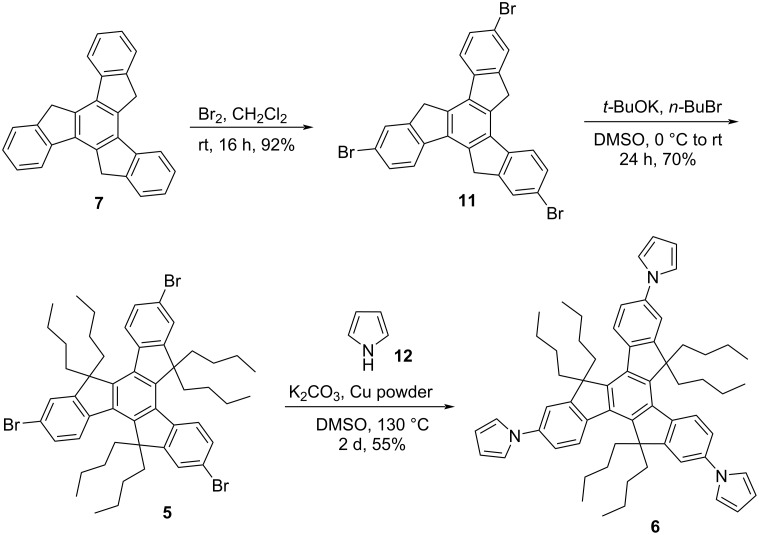
Synthesis of truxene derivative **6** involving Ullmann-type cross-coupling reaction.

Inspired from the available potential applications, and also bearing in mind the applicability of truxene-based materials from future perspective in addition to as a part of our major program on the synthesis of PAHs in general and functionalized truxenes in particular, our voyage for the synthesis of truxene-based heterocyclic systems commence with the construction of tripyrrolotruxene derivative **6** (Scheme 2). Basically, our interest to generate pyrrole-based *C*_3_-symmetric truxene derivatives was because of a range of applications of the pyrrole moiety in diverse fields, for instance, a wide variety of medicinally active as well as smart functional materials possessing the pyrrole as a fundamental subunit [28–30]. Additionally, 3,4-disubstituted pyrrole derivatives are versatile building blocks for the production of diverse bioactive molecules like co-enzyme, alkaloids, porphyrins and other related macrocycles [31–32]. Moreover, polypyrrolic systems are of extensive interest in numerous areas namely optics, medicine, and supramolecular chemistry in addition to the materials sciences and technology. Fascinatingly, in luminescence as well as fluorescence-based sensors, pyrrole derivatives play a vital role as donors in the intramolecular partial charge-transfer cascade to transfer the extra electronic density. Apart from pure affection besides many more reasons for the constant fascination and utility of this beautifully simple yet much effective heteroaromatic system, this unique nitrogen-containing five-membered aromatic pyrrole scaffold will continue to play a significant role in the development of novel anionic receptors [33–34]. Therefore, as outlined in Scheme 2, our synthetic strategy towards the construction of the target pyrrole-based truxene derivative **6** initiated with the acid-catalyzed cyclotrimerization of the easily accessible 1-indanone (**1**) to furnish truxene (**7**) in 80% yield. Having the pristine truxene (**7**) in our hands, it was then subjected to hexaalkylation using *t*-BuOK/*n*-BuBr in DMSO to afford the required hexabutylated truxene derivative **2** in 95% yield (Scheme 2). Next, compound **2** was subjected to nitration followed by reduction to deliver triaminotruxene **4** in excellent yield. Later, compound **4** was subjected to six-fold *N*-allylation in the presence of NaH/allyl bromide to provide the required compound **3** which was directly treated with G-I catalyst (**9**) to afford the desired tripyrrolotruxene **6** in 68% yield (two steps) in a single-pot without further addition of any oxidizing agent, may be due immediate conversion of intermediatory non-aromatized 2,5-dihydro-1*H*-pyrrole derivatives to more stable aromatic pyrrole units (Scheme 2). On the other hand, we have prepared the same compound **6** from the triamine **4** utilizing a three-fold Clauson–Kaas reaction in the presence of 2,5-dimethoxytetrahydrofuran (**10**) under acetic acid reflux conditions (Scheme 3). Alternatively, the pyrrole-based system **6** has also been prepared starting from truxene (**7**) in three steps, involving the bromination using Br_2_/CH_2_Cl_2_ and then subsequent alkylation followed by Ullmann-type copper-mediated cross-coupling reaction in overall good yield (Scheme 4).

**Scheme 5:**
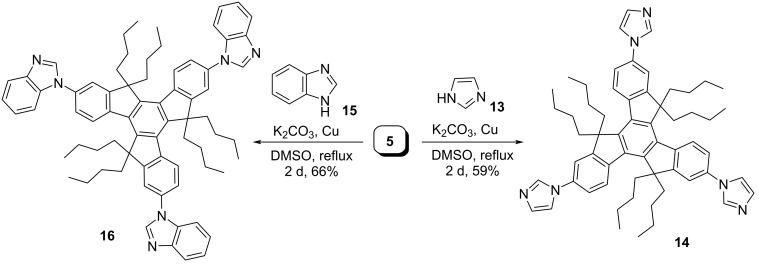
Synthesis of imidazole and benzimidazole containing truxene derivatives **14** and **16**.

**Scheme 6:**
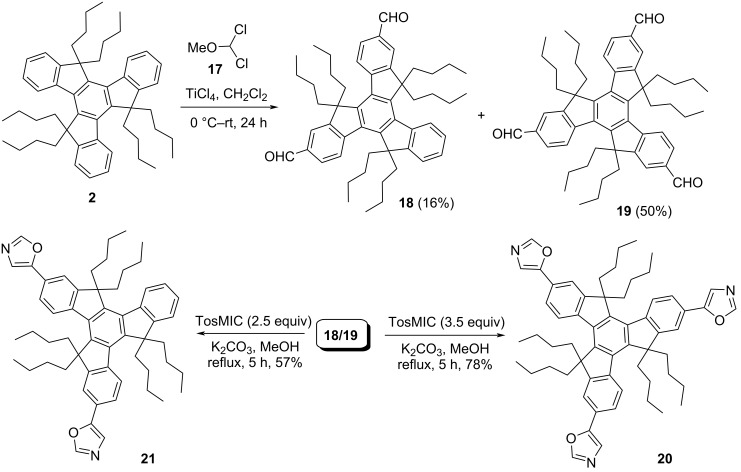
Construction of truxene-based di- and trioxazole derivatives **21** and **20**.

**Scheme 7:**
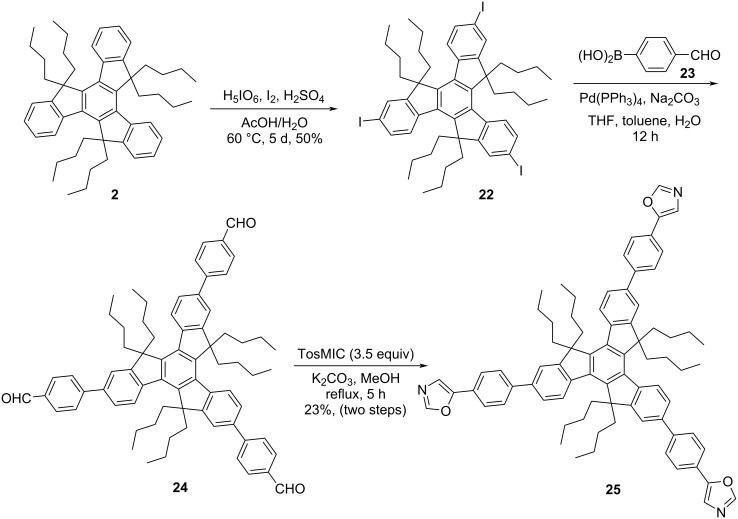
Synthesis of benzene-bridged rings containing trioxazolotruxene system **25**.

On the other hand, imidazole and benzimidazole containing *C*_3_-symmetric truxene-based molecules (**14** and **16**) have also been assembled from the hexaalkylated tribromotruxene **5** by means of Ullmann-type reaction in the presence of copper powder using pristine imidazole (**13**) and benzimidazole (**15**), respectively (Scheme 5). Moreover, we have successfully constructed the *C*_3_-symmetric oxazole containing truxene derivative **20** along with bisoxazole truxene derivative **21** by virtue of Van Leusen reaction as a key step (Scheme 6). To this context, we first treated the hexabutylated derivative **2** with 1,1-dichlorodimethyl ether acting as a formyl source in the presence of Lewis acid such as titanium tetrachloride (TiCl_4_) in Rieche manner to yield the required triformyltruxene **19** as a major product along with a minor amount of diformyltruxene **18** (Scheme 6). Later, both the di- and triformylated truxene systems **18**/**19** were subjected to oxazole formation involving *p*-toluenesulfonylmethyl isocyanide (TosMIC) in Van Leusen fashion to provide the corresponding desired truxene-based oxazole derivatives (Scheme 6). Additionally, to enhance the conjugation which in turn would undoubtedly tune the properties of the truxene-based heterocyclic systems, we also design and synthesized the benzene-bridged oxazole derivative **25** in three steps (Scheme 7). As can be inspected from Scheme 7, our journey in this regard stem from the iodination of **2** using H_5_IO_6_/I_2_/H_2_SO_4_ in acetic acid–water solvent system to afford the desired triiodotruxene derivative **22** in 50% yield. Furthermore, Suzuki–Miyaura cross-coupling reaction of **22** with 4-formylphenylboronic acid (**23**) furnished the triformyltruxene **24** (Scheme 7). Finally, the crude product **24** was directly treated with TosMIC under Van Leusen oxazole conditions to provide the required product **25** in 23% yield (two steps) as depicted in Scheme 7.

### Absorption and emission spectra

**Figure 1:**
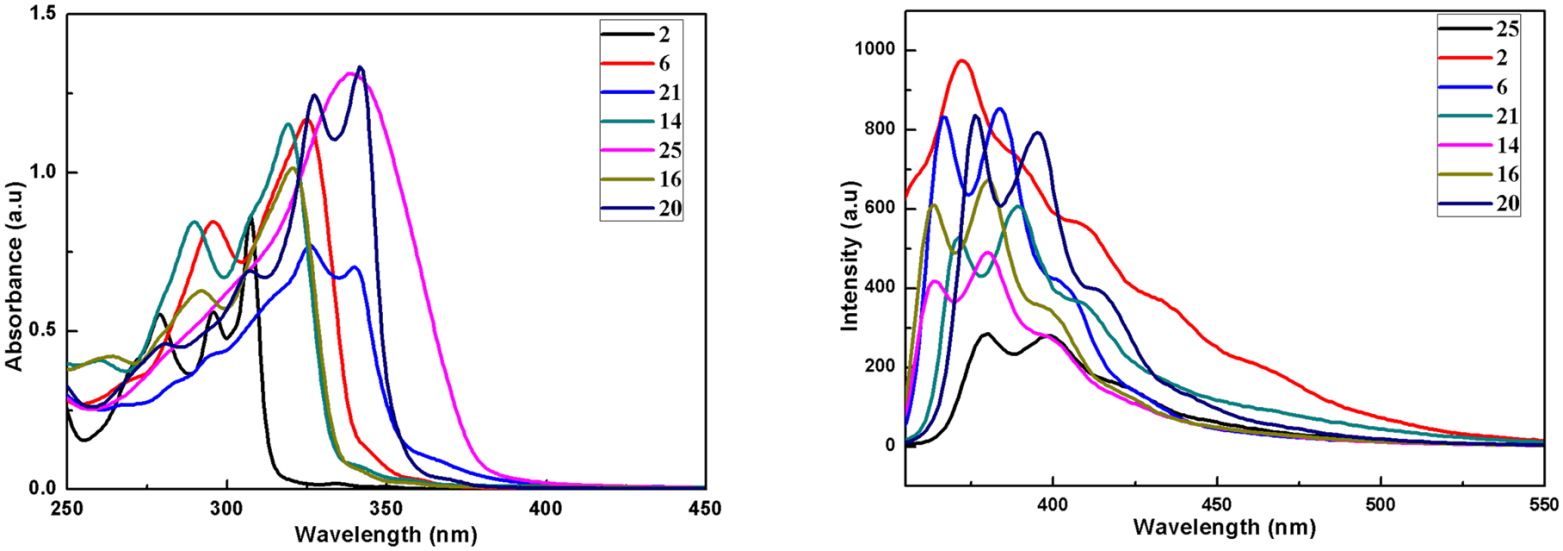
Normalized absorption (left); fluorescence spectra (right) of the synthesized truxene derivatives (7.04 × 10^−6^ M concentration) in chloroform.

The UV–vis absorption as well as fluorescence spectra of the newly synthesized truxene derivatives **2**, **6**, **14**, **16**, **20**, **21** and **25** were recorded in chloroform using a 7.04 × 10^−6^ molar concentration of the solution at room temperature (Figure 1). As can be inspected from Figure 1, these truxene systems exhibit absorption bands between 278–342 nm corresponding to the π–π* transitions. The absorption spectra of the parent hexa-alkylated truxene derivative **2** exhibited a strong band at 307.27 nm along with two more bands at ca. 295.62 nm, and 278.44 nm. On the other hand, a strong band with absorption maxima at 325.48 and a less intense band at 295.62 nm were observed for tripyrrolotruxene **6**. Similarly, two bands for example at 318.91 nm and 290.55 nm were noticed in the case of imidazole containing truxene derivative **14**. Interestingly, to our expectation, in both the cases, i.e., in truxenes **6** and **14**, we found almost similar types of spectrum possessing red-shift absorption maxima as compared to the parent compound **2**, may be due to increased conjugation in these truxene systems with the incorporation of three heterocyclic units in each case (Figure 1). Moreover, the absorption spectrum of compound **16** exhibited slightly red-shifted maxima at ca. 320.41 nm and 291.07 nm in addition to an extra band at 263.22 nm due to the fusion of three benzene rings onto the imidazole moieties. In sharp contrast, more red-shifted absorption bands at 341.64 nm and 326.99 nm along with two shoulders at ca. 306.75 nm, and 279.94 nm were found with truxene having oxazole moiety **20** in its structure. As expected, in the case of dioxazole truxene system **21**, the bands were noticed at slightly lower wavelengths (340.13 nm and 325.96 nm) as compared to truxene **20** having three oxazole units, due to lack of one conjugated oxazole subunit. Finally and more surprisingly, compound **25** exhibited only a single broad band at ca. 339.10 nm as can be inspected from Figure 1. To our expectation, as can be seen from an inspection of Figure 1, emission spectra of all the synthesized truxenes lies at longer wavelengths as compared to the absorption spectra for the corresponding truxene derivatives. The fluorescence spectrum of the parent truxene **2** exhibit a broad band at ca. 371.88 nm while as all the other truxenes displayed two bands in the range of 364–399 nm with a very small shoulder in each case, which shows well-defined vibronic features (Figure 1). The exact values of the fluorescence maxima for these compounds are as follow; **6** (366.47 and 383.73), **14** (363.50 and 379.75), **16** (363.50 and 380.25), **20** (370.32 and 395.54), **21** (371.37 and 389.64), and **25** (379.26 and 398.52).

## Conclusion

In summary, the scope of applicability of truxene and its congeners is continuously exploding day-by-day, thanks to its easy construction, and solubility in common organic solvents in addition to remarkable structural modifications of the truxene framework. Interestingly, the area of truxene is now fully matured, scientist’s imagination is the only limit for designing novel truxene-based functional materials and the opportunities are now open. Herein, we have assembled diverse truxene scaffolds containing heterocyclic systems involving trimerization, ring-closing metathesis, Clauson–Kaas and Van Leusen reactions as vital steps. Moreover, we have revealed the preliminary photophysical properties (absorption and emission spectra) of the newly synthesized molecules and the detailed studies for these systems would be published in due course. We are pretty sure that this particular work may offer guidance for further expansion of these materials with enhanced potential for optoelectronic applications and other, if any in material science and technology.

## Experimental

**General:** All commercially available reagents were used without further purification and purchased from Sigma-Aldrich, Alfa Aesar, TCI, GLR, Avera or Spectrochem. Solvents were purified according to reported standard procedures. Pyrrole was used after distillation. Analytical thin-layer chromatography (TLC) was performed on aluminium plates coated with silica gel by using a suitable mixture of EtOAc and petroleum ether for development. Column chromatography was performed by using silica gel (100–200 mesh) with an appropriate mixture of EtOAc and petroleum ether. Characterization of the intermediates and final compounds were performed using ^1^H and ^13^C NMR, mass spectrometry and IR spectroscopy. FTIR spectra of the compounds were recorded on an Aligent FTIR spectrometer (ATR module of Cary 630 FTIR, Agilent Technologies) from 600 to 4000 cm^−1^. UV–vis spectra were recorded on a UV-1800 Shimadzu spectrophotometer and the fluorescence spectra were recorded on an Agilent Carry Eclipse spectrofluorimeter.

**Synthesis of 10,15-dihydro-5*H*-diindeno[1,2-*a*:1',2'-*c*]fluorene (7):** Truxene scaffold **7** was prepared according to a literature reported procedure [35]. l-Indanone (**1**, 6.8 g, 51.4 mmol) was added to a mixture of 60 mL acetic acid and 30 mL concentrated HCl, then the reaction mixture was stirred for 16 h at 100 °C. The solid precipitate was obtained by pouring the reaction mixture into crushed ice, washing with water, acetone followed by dichloromethane gave a white powder (4.7 g, 80%).

**Synthesis of 5,5,10,10,15,15-hexabutyl-10,15-dihydro-5*H*-diindeno[1,2-*a*:1',2'-*c*]fluorene (2):** Truxene (**7**, 4 g, 11.68 mmol), DMSO (35 mL), and *t*-BuOK (11.79 g, 105.12 mmol) were mixed in a two-necked round bottom flask (100 mL) under nitrogen atmosphere. The flask was cooled to 0 °C and stirred viciously after that *n*-BuBr (11.30 mL, 105.12 mmol) was slowly added to the flask, and the stirring was continued for 24 h at room temperature (rt), till the completion of the reaction (TLC monitoring). Then, the reaction was quenched with water and the crude product was extracted with ethyl acetate. The combined organic layer was washed with water (100 mL × 2) and dried over Na_2_SO_4_. After evaporation of the solvents, the residue was passed through silica gel to give the final product as a white powder (7.5 g, 95%). The ^1^H NMR spectrum was perfectly matching with the previously reported one [36].

**Synthesis of 5,5,10,10,15,15-hexabutyl-2,7,12-trinitro-10,15-dihydro-5*H*-diindeno[1,2-*a*:1',2'-*c*]fluorene (8):** To a solution of compound **2** (3.5 g, 5.15mmol) in dichloromethane (50 mL), conc. HNO_3_ (6.23 mL, 103 mmol) was slowly added and the resulting mixture was stirred for 2 h at 0 °C followed by 24 h at room temperature. Next, the reaction mixture was poured into ice cold water and neutralized with NaOH solution. Then the organic layer was extracted with ethyl acetate (50 mL × 3), dried over Na_2_SO_4_ and the crude product was purified with column chromatography to deliver the required compound **8** as yellow solid (4.15 g, 99%); *R*_f_ = 0.52 (10% EtOAc/petroleum ether); ^1^H NMR (300 MHz, CDCl_3_) δ 8.53 (d, *J* = 8.7 Hz, 3H), 8.37 (d, *J* = 9 Hz, 6H), 2.99–2.90 (m, 6H), 2.21–2.23 (m, 6H), 0.99–0.82 (m, 12H), 0.58-0.52 (m, 30H); ^13^C NMR (75 MHz, CDCl_3_) δ 154.86, 149.65, 147.08, 145.20, 137.48, 124.91, 122.66, 117.60, 56.57, 36.44, 26.54, 22.63, 13.71; IR (KBr): 2957, 2925, 2858, 1742, 1594, 1517, 1458 cm^−1^; MS (*m*/*z*): 814.00.

**Synthesis of 5,5,10,10,15,15-hexabutyl-10,15-dihydro-5*H*-diindeno[1,2-*a*:1',2'-*c*]fluorene-2,7,12-triamine (4):** To a 100 mL round bottom flask, SnCl_2_·2H_2_O (16.63 g, 73.5 mmol), compound **8** (2 g, 2.45 mmol) and dry ethyl acetate (60 mL) were added. Later, the resulting mixture was refluxed for 8 h and after completion of the reaction (TLC monitoring), the solution was quenched with aqueous saturated sodium bicarbonate solution within an ice bath. Then the reaction mixture was filtered and the organic layer was extracted with ethyl acetate (40 mL × 3), dried over Na_2_SO_4_ and after removing the solvent compound **4** was obtained (1.75 g, 99%); *R*_f_ = 0.36 (30% EtOAc/petroleum ether); ^1^H NMR (300 MHz, CDCl_3_) δ 8.08 (d, *J* = 8.4 Hz, 3H), 6.78 (s, 3H) 6.70 (d, *J* = 8.1 Hz, 3H), 3.69 (b–NH, 6H), 2.91–2.81 (m, 6H), 1.96–1.87 (m, 6H), 0.94–0.60 (m, 12H), 0.56–0.42 (m, 30H); ^13^C NMR (75 MHz, CDCl_3_) δ 155.72, 144.69, 141.19, 138.07, 132.30, 125.41, 113.23, 109.04, 55.05, 36.85, 26.50, 22.95, 13.91; IR (KBr): 3369, 3012, 2953, 2922, 2855, 1619, 1584, 1485 cm^−1^; MS (*m*/*z*): 724.66.

**Synthesis of *N*^2^*,N*^2^*,N*^7^*,N*^7^*,N*^12^*,N*^12^-hexaallyl-5,5,10,10,15,15-hexabutyl-10,15-dihydro-5*H*-diindeno[1,2-*a*:1',2'-*c*]fluorene-2,7,12-triamine (3):** To a suspension of sodium hydride (132 mg, 5.52 mmol) in dry DMF (5 mL), was added triamine derivative **4** (500 mg, 0.69 mmol) in DMF (5 mL) and allyl bromide (0.48 mL, 5.52 mmol) at 0 °C under nitrogen atmosphere. The mixture was stirred at rt for 12 h. After completion of the reaction (TLC monitoring), the reaction mixture was quenched with saturated NH_4_Cl and the aqueous layer was extracted with EtOAc (50 mL × 3), dried over Na_2_SO_4_. The solvent was removed under reduced pressure and the crude product was directly used for the next step.

**Synthesis of 1,1',1''-(5,5,10,10,15,15-hexabutyl-10,15-dihydro-5*H*-diindeno[1,2-*a*:1',2'-*c*]fluorene-2,7,12-triyl)tris(1*H*-pyrrole) (6): Method A:** The solution of crude product **3** in dry dichloromethane (15 mL) was degassed with nitrogen for 15 min. After adding the Grubbs’ catalyst (15 mol %), the stirring was continued for 12 h at the same temperature. Next, the solvent was removed under reduced pressure followed by silica gel column chromatography (5% EtOAc/petroleum ether) provided the required product **6** (410 mg, 68%); *R*_f_ = 0.60 (5% ethyl acetate/petroleum ether); ^1^H NMR (400 MHz, CDCl_3_) δ 8.39 (d, *J* = 8 Hz, 3H), 7.47–7.45 (m, 6H), 7.26–7.25 (m, 6H), 6.43–6.41 (m, 6H), 3.02–2.91 (m, 6H), 2.15–2.08 (m, 6H), 0.95–0.89 (m, 12H), 0.49–0.46 (m, 30H); ^13^C NMR (100 MHz, CDCl_3_) δ 155.42, 144.58, 139.38, 137.83, 137.70, 125.49, 119.39, 118.48, 114.03, 110.51, 110.33, 55.84, 36.76, 26.54, 22.83, 13.85; IR (KBr): 2953, 2922, 2855, 1962, 1608, 1497 cm^−1^; MS (*m*/*z*): 874.95.

**Method B:** Triaminotruxene **4** (300 mg, 0.41 mmol) and 2,5-dimethoxytetrahydrofuran (**10**, 273.77 mg, 2.05 mmol**)** were dissolved in acetic acid (5 mL) and the reaction mixture was heated under reflux conditions for 4 h in nitrogen atmosphere. After completion of the reaction (TLC monitoring), the reaction mixture was cooled to room temperature and quenched with saturated NH_4_Cl solution. Then, the aqueous layer was extracted with EtOAc (30 mL × 3) and the organic portion was dried over Na_2_SO_4_. The solvent was removed at reduced pressure and the crude product was purified by silica gel column chromatography (5% EtOAc/petroleum ether) to afford tripyrrolotruxene **6** (155 mg, 43%) as a brown solid. The spectral data matched with the above data (method A).

**Method C:** Tribromotruxene derivative **5** (400 mg, 0.43 mmol), pyrrole (**12**, 102.5 mg, 1.50 mmol), K_2_CO_3_ (6.5 equiv), and copper power (3.5 equiv) were dissolved in dry DMSO (10 mL) and the reaction mixture was heated at 130 °C under nitrogen atmosphere for 2 days. After completion of the reaction (TLC monitoring), the reaction mixture was cooled to room temperature and the aqueous layer was extracted with EtOAc (10 mL × 3). Organic portion was dried over Na_2_SO_4_ and the solvent was removed at reduced pressure. Then, the crude product was purified by silica gel column chromatography (5% EtOAc/petroleum ether) to give the required product **6** (208 mg, 55%) as a brown solid. Spectral data was matched with the above compound obtained by method A.

**Synthesis of 2,7,12‐tribromo‐10,15‐dihydro‐5*H*‐diindeno[1,2‐*a*;1′,2′‐*c*]fluorene (11):** Compound **11** was prepared according to a literature reported procedure [37]. Bromine (2 mL, 29.2 mmol) was added dropwise to a suspension of truxene **7** (2 g, 5.84 mmol) in CH_2_Cl_2_ (15 mL) at rt. The reaction mixture was stirred for 16 h at the same temperature and the excess of bromine was removed by bubbling N_2_ through the solution. Then the solid was filtered, washed with CH_2_Cl_2_, acetone, and finally with ethyl acetate to give **11** (3.13 g, 92%) as a yellow solid.

**Synthesis of 2,7,12-tribromo-5,5,10,10,15,15-hexabutyl-10,15-dihydro-5*H*-diindeno[1,2-*a*:1',2'-*c*]fluorene (5):** Compound **11** (1.5 g, 2.59 mmol), dimethyl sulfoxide (20 mL), and potassium *tert-*butoxide (2.61 g, 23.31 mmol) were mixed in a three-necked round bottom flask (100 mL) under nitrogen atmosphere. The flask was cooled to 0 °C and stirred viciously. *n*-Butyl bromide (2.50 mL, 23.31 mmol) was then slowly added to the flask, and the reaction was returned to rt. The reaction was quenched with water and the organic layer was extracted with ethyl acetate. The combined organic layer was washed with water (50 mL × 3) and dried over Na_2_SO_4_. After evaporation of the solvent, the residue was passed through silica gel to provide the final product **5** as a white powder (1.10 g, 70%). The ^1^H NMR spectrum of compound **5** was matched with the literature reported (see Supporting Information File 1) [36]. ^1^H NMR (300 MHz) δ 8.21 (d, *J* = 8.6 Hz, 3H), 7.58 (s, 3H), 7.54 (d, *J* = 8.6 Hz, 3H), 2.93–2.83 (m, 6H), 2.11–2.01 (m, 6H), 0.99–0.84 (m, 12H), 0.48 (t, *J* = 8 Hz, 30H).

**Synthesis of 1,1',1''-(5,5,10,10,15,15-hexabutyl-10,15-dihydro-5*H*-diindeno[1,2-*a*:1',2'-*c*]fluorene-2,7,12-triyl)tris(1*H*-imidazole) (14):** A round-bottomed flask was loaded with compound **5** (800 mg, 0.87 mmol), K_2_CO_3_ (1.44 g, 10.4 mmol), imidazole (**13**, 0.35 g, 5.22 mmol), and Cu powder (0.33 g, 5.22 mmol) in DMSO (10 mL) and the reaction mixture was heated at 185 °C for 2 days. Then the organic layer was extracted with ethyl acetate and washed several times with water to remove the DMSO. The resulting organic solution was dried over Na_2_SO_4_, and then the solvent was removed under vacuum which on purification furnished the desired compound **14** (450 mg, 59%) as a yellow solid; *R*_f_ = 0.60 (8% MeOH/petroleum ether); ^1^H NMR (400 MHz, CDCl_3_) δ 8.39 (d, *J* = 8 Hz, 3H), 7.96 (s, 3H), 7.40 (d, *J* = 12 Hz, 9H), 7.22 (s, 3H), 2.94–2.88 (m, 6H), 2.10–2.05 (m, 6H), 0.92–0.83 (m, 12H), 0.43–0.40 (t, *J* = 8 Hz, 30H); ^13^C NMR (101 MHz, CDCl_3_) δ 155.71, 145.45, 139.12, 137.68, 136.12, 135.67, 130.49, 125.74, 119.63, 118.33, 115.05, 56.05, 36.68, 26.54, 22.76, 13.81; IR (KBr): 2953, 2921, 2855, 1609, 1497 cm^−1^; MS (*m*/*z*): 877.79.

**Synthesis of 1,1',1''-(5,5,10,10,15,15-hexabutyl-10,15-dihydro-5*H*-diindeno[1,2-*a*:1',2'-*c*]fluorene-2,7,12-triyl)tris(1*H*-benzo[*d*]imidazole) (16):** A round-bottomed flask was loaded with compound **5** (500 mg, 0.54 mmol), K_2_CO_3_ (0.90 g, 6.48 mmol), benzimidazole (**15**, 387.0 mg, 3.24 mmol), and Cu powder (208 mg, 3.24 mmol) in DMSO (10 mL) and the reaction mixture was heated at 185 °C for 2 days. Then the organic layer was extracted with ethyl acetate and washed several times with water to remove the DMSO. The resulting organic solution was dried over Na_2_SO_4_, and then the solvent was removed under vacuum which on purification delivered the desired compound **16** (0.37 g, 66%) as an off white solid; ^1^H NMR (400 MHz, CDCl_3_) δ 8.58 (d, *J* = 8 Hz, 3H), 8.31 (s, 3H), 7.97–7.95 (m, 3H), 7.67 (d, *J* = 4 Hz, 6H), 7.64–7.61 (m, 3H), 7.43–7.41 (m, 6H) 3.08–3.02 (m, 6H), 2.26–2.19 (m, 6H), 1.06–0.99 (m, 12H), 0.58–0.54 (t, *J* = 8 Hz, 30H); ^13^C NMR (101 MHz, CDCl_3_) δ 155.84, 145.86, 144.20, 142.41, 139.55, 137.86, 135.11, 133.75, 125.95, 123.84, 123.00, 122.13, 120.83, 117.72, 110.58, 108.14, 56.17, 36.74, 26.67, 22.87, 13.91; IR (KBr): 2952, 2920, 2853, 1605, 1490, 1454 cm^−1^; MS (*m*/*z*): 1027.00.

## Supporting Information

File 1 Additional general procedures, experimental and analytical data as well as copies of NMR spectra.
