# Supplementary material for: Design, synthesis and photophysical properties of novel star-shaped truxene-based heterocycles utilizing ring-closing metathesis, Clauson–Kaas, Van Leusen and Ullmann-type reactions as key tools
Source: Beilstein J Org Chem. 2021 Jun 2;17:1374–84. doi: 10.3762/bjoc.17.96 (PMC8182681; doi:10.3762/bjoc.17.96)
Supplement: File 1 — Additional general procedures, experimental and analytical data as well as copies of NMR spectra. [file Beilstein_J_Org_Chem-17-1374-s001.pdf]

BEILSTEIN JOURNAL OF ORGANIC CHEMISTRY

Supporting Information

for

Design, synthesis and photophysical properties of novel star-shaped truxene-based heterocycles utilizing ring-closing metathesis, Clauson–Kaas, Van Leusen and Ullmann-type reactions as key tools

Shakeel Alvi and Rashid Ali

Beilstein J. Org. Chem. **2021,** *17,* 1374–1384. doi:10.3762/bjoc.17.96

Additional general procedures, experimental and analytical data as well as copies of NMR spectra

License and Terms: This is a supporting information file under the terms of the Creative Commons Attribution License (https://creativecommons.org/licenses/by/4.0). Please note that the reuse, redistribution and reproduction in particular requires that the author(s) and source are credited and that individual graphics may be subject to special legal provisions.
The license is subject to the *Beilstein Journal of Organic Chemistry* terms and conditions: (https://www.beilstein-journals.org/bjoc/terms)

Table of contents

General procedure for formation of compound **18** and **19** S3

Synthesis of **18** S3

Synthesis of **19** S3

General procedure for the formation of truxene-based oxazole derivatives **20, 21** and **25** S3

Synthesis of **21** S4

Synthesis of **20** S4

Synthesis of **25** S4

Synthesis of **22** S4

Synthesis of **24** S5

^{1}H and ^{13}C NMR spectrum of compound **8** S6

Mass spectra of compound **8** S7

IR spectrum of compound **8** S7

^{1}H and ^{13}C NMR spectrum of compound **4** S8

Mass spectra of compound **4** S9

IR spectrum of compound **4** S10

^{1}H NMR spectrum of compound **6** S10

^{13}C NMR spectrum of compound **6** S11

Mass spectra of compound **6** S12

IR spectrum of compound **6** S13

^{1}H NMR spectrum of compound **5** S14

^{1}H and ^{13}C NMR spectrum of compound **14** S15

Mass spectra of compound **14** S15

IR spectrum of compound **14** S16

^{1}H NMR spectrum of compound **16** S16

^{13}C NMR spectrum of compound **16** S17

Mass spectra of compound **16** S17

IR spectrum of compound **16** S18

^{1}H NMR spectrum of compound **18** S18

^{1}H and ^{13}C NMR spectrum of compound **21** S19

Mass spectra of compound **21** S20

IR spectrum of compound **21**	S20
^{1}H and ^{13}C NMR spectrum of compound **20**	S21
Mass spectra of compound **20**	S22
IR spectrum of compound **20**	S22
^{1}H and ^{13}C NMR spectrum of compound **25**	S23
Mass spectra of compound **25**	S24
IR spectra of compound **25**	S24
^{1}H NMR spectrum of compound **22**	S25
References	S25

General procedure for formation of compound 18 and 19

Under nitrogen atmosphere, **2** (3 g, 4.41 mmol) was dissolved in dried CH_2Cl_2 (10 mL) at 0 °C. A batch of $TiCl_4$ (5.87, 52.92 mmol) in CH_2Cl_2 was added dropwise during 30 min with sufficient stirring. The purple reaction mixture was stirred for another 1 h at 0 °C, and then a solution of CH_3OCHCl_2 **17** (5 mL, 52.92 mmol) in 10 mL of CH_2Cl_2 was added dropwise over 10 min at 0 °C. The mixture was allowed to warm slowly and stirred for 24 h at room temperature. After completion of the reaction (TLC monitoring), the purple mixture was poured into crushed ice and the aqueous layer was extracted with ethyl acetate. The combined organic fractions were washed with water and brine and finally dried over Na_2SO_4. The solution was concentrated under reduced pressure, and the residue was purified with column chromatography (silica gel, ethyl acetate/hexanes) to provide the desired formylated truxene derivatives **18** and **19**.

Synthesis of 5,5,10,10,15,15-hexabutyl-10,15-dihydro-5*H*-diindeno[1,2-*a*:1',2'-*c*]fluorene-2,7-dicarbaldehyde (18): Yellow solid; yield = 16% (518 mg, starting from 3 g of **2**); R_f = 0.63 (15% ethyl acetate/petroleum ether). The ^{1}H NMR spectrum of this compound was matched with the reported one [1].

Synthesis of 5,5,10,10,15,15-hexabutyl-10,15-dihydro-5*H*-diindeno[1,2-*a*:1',2'-*c*]fluorene-2,7,12-tricarbaldehyde (19): Yellow solid; yield = 50% (1.70 g, starting from 3 g of **2**); R_f = 0.53 (15% ethyl acetate/petroleum ether) [2].

General procedure for the formation of truxene-based oxazole derivatives 20, 21 and 25:

In a two-necked round bottom flask (50 mL), the aldehydes **18**/**19**/**24** (1.0 equiv) were dissolved in dry methanol (10 mL) then TosMIC (2.5/3.5 equiv.) and K_2CO_3 (3.0 equiv for each formyl group) were added portion wise to the reaction mixture. The reaction mixture was heated at 70 °C for 5 h. After the completion of the reaction (TLC monitoring), the mixture was cooled to rt and methanol was removed under reduced pressure. The reaction mixture was extracted with EtOAc (3 × 15 mL) and the combined organic layer was washed with both water as well as brine solution and dried with Na_2SO_4. The solvent was removed on a rotavapor and the crude products were purified by silica gel column chromatography using the appropriate mixtures of EtOAc/petroleum ether to afford the corresponding desired oxazole derivatives.

Synthesis of 5,5'-(5,5,10,10,15,15-hexabutyl-10,15-dihydro-5*H*-diindeno[1,2-*a*:1',2'-*c*]fluorene-2,7-diyl)bis(oxazole) (21): Off white solid; yield = 57% (131 mg, starting from 210 mg of di-aldehyde **18**); R_f = 0.62 (20% ethyl acetate/petroleum ether); ^{1}H NMR (400 MHz, $CDCl_3$): δ 8.46–8.38 (m, 3H), 8.00 (s, 2H), 7.75–7.73 (m, 4H), 7.49 (d, *J* = 4 Hz, 3H), 7.43–7.40 (m, 2H), 3.07–2.92 (m, 6H), 2.19–2.11 (m, 6H), 0.95–0.88 (m, 12H) , 0.50–0.43 (m, 30H); ^{13}C NMR (101 MHz, $CDCl_3$): δ 154.53, 154.40, 153.50, 152.03, 150.36, 145.74, 145.68, 145.30, 140.86, 140.79, 139.91, 138.80, 137.93, 137.78, 126.74, 126.20 , 125.81, 125.14, 125.03, 124.74, 122.73, 122.36, 121.46, 118.01, 117.97, 55.86, 55.79, 55.66, 36.71, 36.58, 26.56, 26.50, 22.84, 22.82, 13.81; IR (KBr) 2945, 2923, 2857, 1687, 1604, 1459 cm^{-1}. MS (m/z): 813.40.

Synthesis of 5,5',5''-(5,5,10,10,15,15-hexabutyl-10,15-dihydro-5*H*-diindeno[1,2-*a*:1',2'-*c*]fluorene-2,7,12-triyl)tris(oxazole) (20): Pale yellow solid; yield = 78% (303 mg, starting from 300 mg of tri-aldehyde **19**); R_f = 0.41 (20% ethyl acetate/petroleum ether); ^{1}H NMR (500 MHz, $CDCl_3$): δ 8.38 (d, *J* = 8.0 Hz, 3H), 7.93 (bs, 3H), 7.69 (bs, 6H), 7.42 (bs, 3H), 2.94–2.90 (m, 6H), 2.12–2.08 (m, 6H), 0.88–0.80 (m, 12H), 0.38 (t, *J* = 4.0 Hz, 30H); ^{13}C NMR (125 MHz, $CDCl_3$): δ 154.40, 151.94, 150.42, 145.86, 140.58, 138.02, 126.00, 125.12, 122.83, 121.57, 118.01, 55.88, 36.69, 26.56, 22.82, 13.83; IR (KBr) 3119, 2954, 2924, 2856, 1699, 1601, 1473 cm^{-1}; MS (m/z): 880.6.

Synthesis of 5,5',5''-((5,5,10,10,15,15-hexabutyl-10,15-dihydro-5*H*-diindeno[1,2-*a*:1',2'-*c*]fluorene-2,7,12-triyl)tris(benzene-4,1-diyl))tris(oxazole) (25): Yellow solid; = 23% (96 mg, starting from **22**) two steps yield; R_f = 0.34 (40% ethyl acetate/petroleum ether); ^{1}H NMR (400 MHz, $CDCl_3$): δ 8.49 (d, *J* = 8 Hz, 3H), 7.96 (d, *J* = 4 Hz, 3H), 7.97–7.74 (m, 18H), 7.44 (s, 3H), 3.10–3.03 (m, 6H), 2.26-2.18 (m, 6H), 1.01-0.89 (m, 12H), 0.51–0.47 (t, *J* = 8 Hz, 30H); ^{13}C NMR (100 MHz, $CDCl_3$): δ 154.47, 151.48, 150.54, 145.59, 141.43, 140.03, 138.17, 138.12, 127.57, 127.47, 126.59, 125.10, 124.91, 121.60, 120.47, 117.70, 108.17, 55.83, 36.84, 26.63, 22.92, 13.89; IR (KBr) 2953, 2923, 2855, 1686, 1602, 1474 cm^{-1}; MS (m/z): 1108.01.

Synthesis of 5,5,10,10,15,15-hexabutyl-2,7,12-triiodo-10,15-dihydro-5*H*-diindeno[1,2-*a*:1',2'-*c*]fluorene (22): A solution of compound **2** (2.5 g, 3.68 mmol) in 10 mL of solvent mixture ($CH_3COOH/H_2SO_4/H_2O$) (100:40:3) was heated at 60 °C with vigorous stirring, followed by addition of $CHCl_3$ (3 mL), H_5IO_6 (1.0 g, 3.68 mmol), and I_2 (2.80 g, 11.04 mmol). Then the reaction mixture was stirred at 80 °C under nitrogen atmosphere. A small amount of H_5IO_6 was added to the mixture until the completion of the reaction (TLC monitoring), the

mixture was cooled to rt, and 150 mL of water was added it. The brown precipitate was filtered and purified by recrystallization three times from ethanol to afford **22** (2 g, 50%) as a white solid. The ^{1}H NMR spectrum of this compound was matched with the reported one [3].

Synthesis of 4,4',4''-(5,5,10,10,15,15-hexabutyl-10,15-dihydro-5*H*-diindeno[1,2-*a*:1',2'-*c*]fluorene-2,7,12-triyl)tribenzaldehyde (24): A solution of **22** (400 mg, 0.37 mmol) in 30 mL of THF/toluene/water (1:1:1), were added Na_2CO_3 (9.0 equiv) and 4-formylphenylboronic acid (**23**, 5.0 equiv). The reaction mixture was degassed with nitrogen for 15–20 min. The $Pd(PPh_3)_4$ (15 mol %) was then added to the reaction mixture and heated at 100 °C for 12 h. After completion (TLC monitoring), the reaction mixture was diluted with water and the organic layer was extracted with CH_2Cl_2. The solvent was removed through rotavapor and the crude Suzuki product was directly used for further reaction as such without purification.

^{1}H NMR spectrum of compound 8

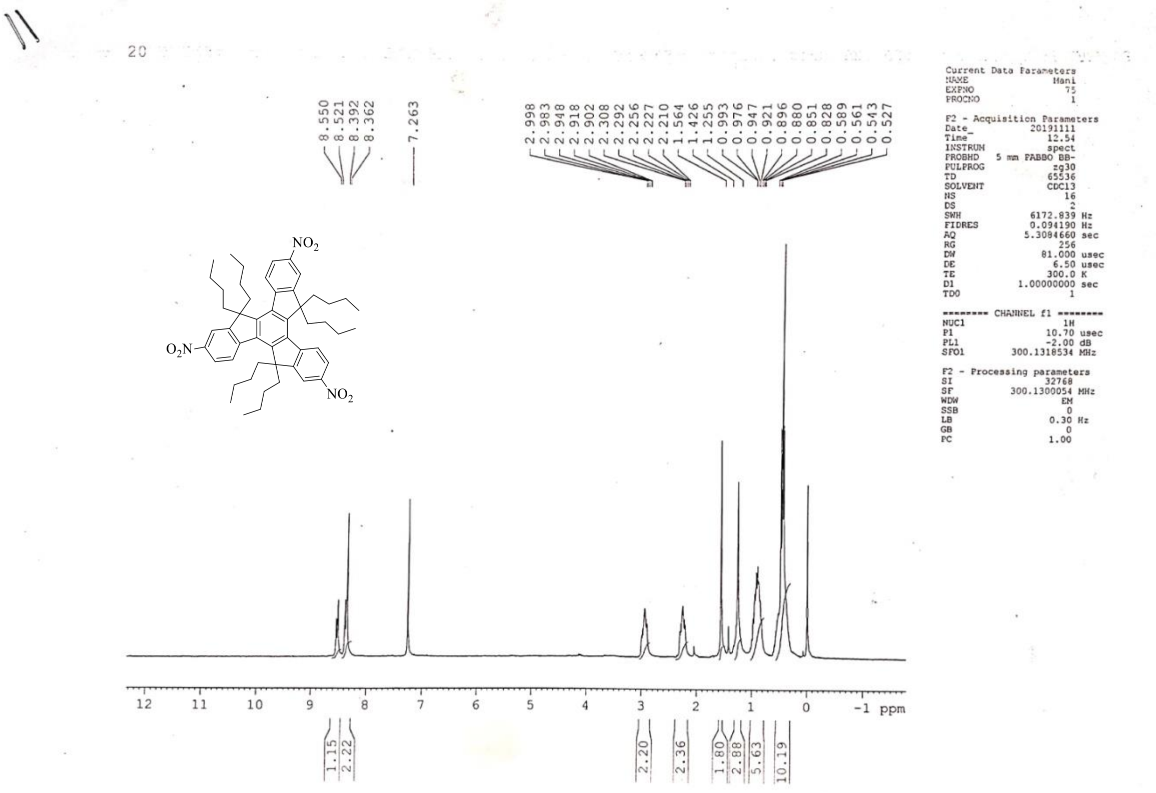

^{13}C NMR spectrum of compound 8

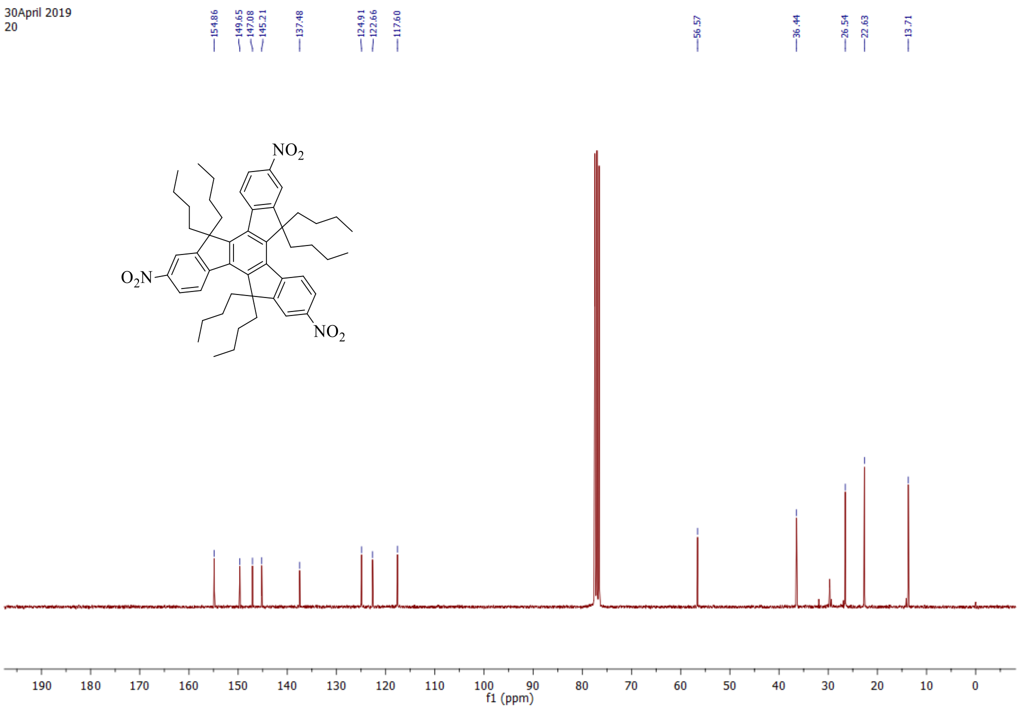

Mass spectra of compound 8

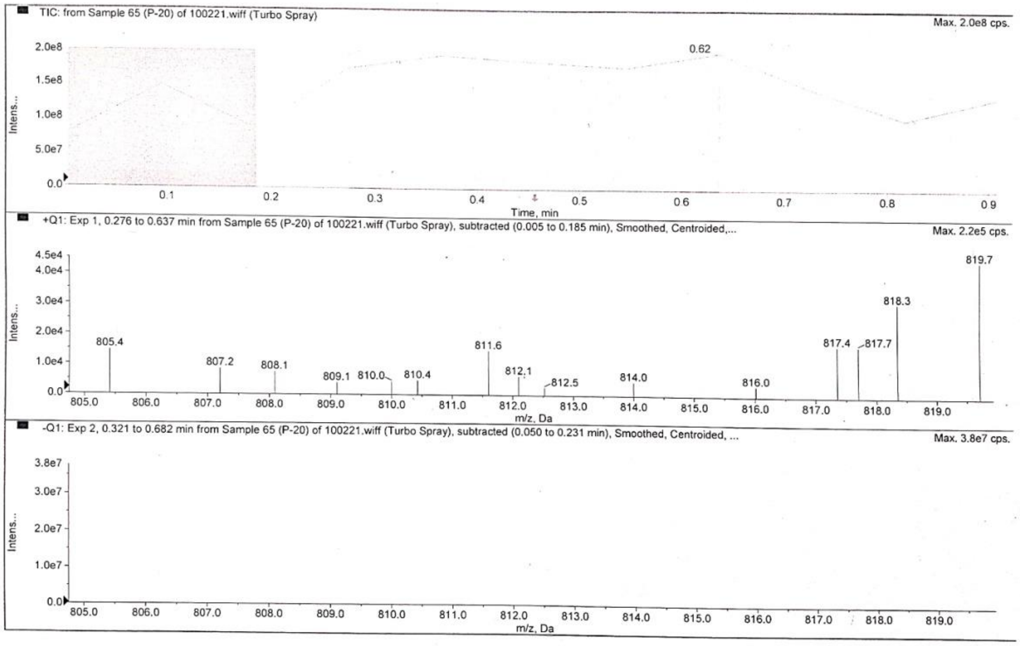

IR spectrum of compound 8

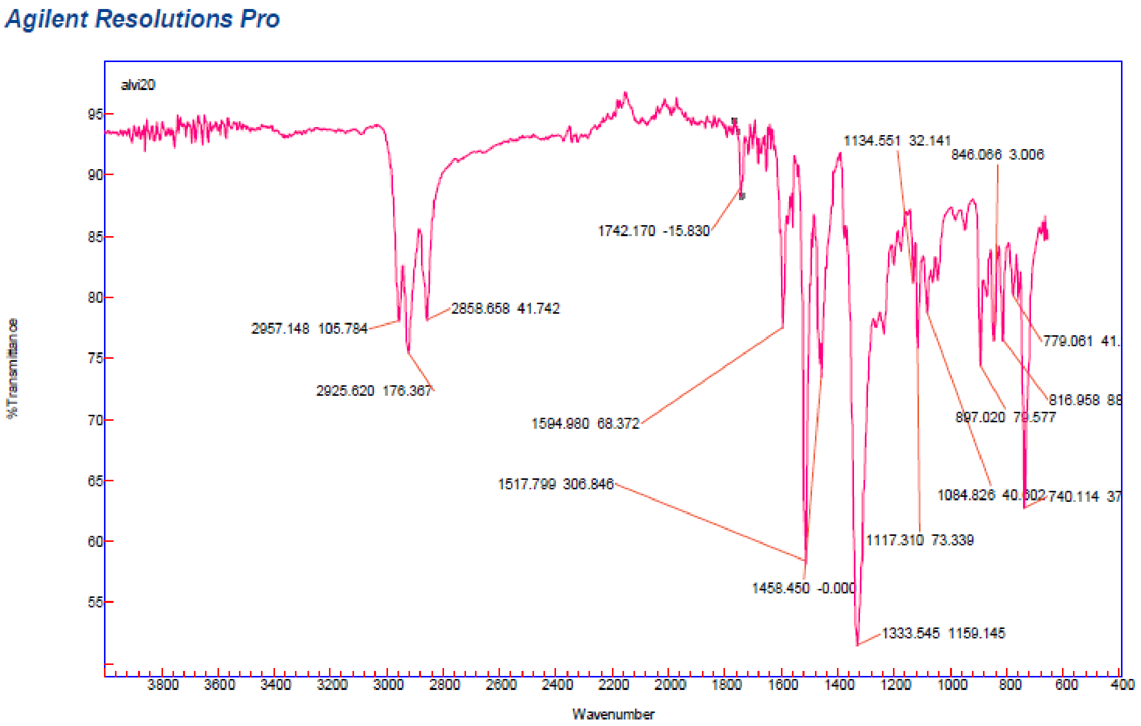

^{1}H NMR spectrum of compound 4

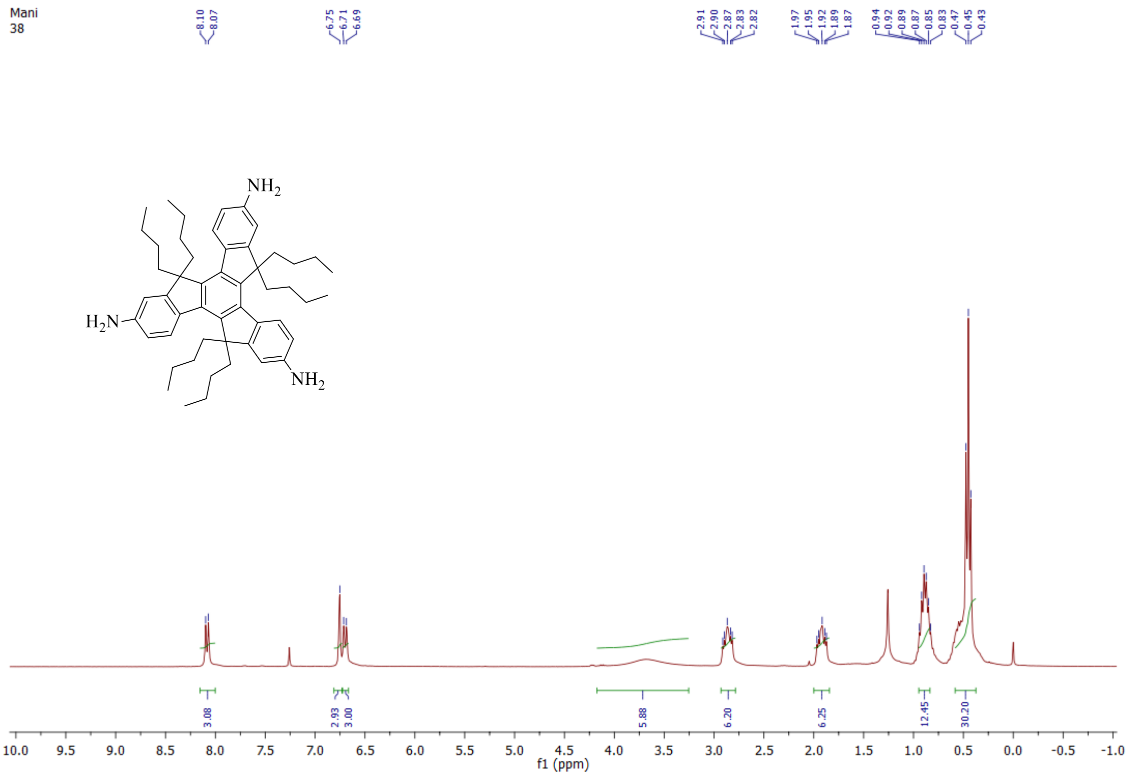

^{13}C NMR spectrum of compound 4

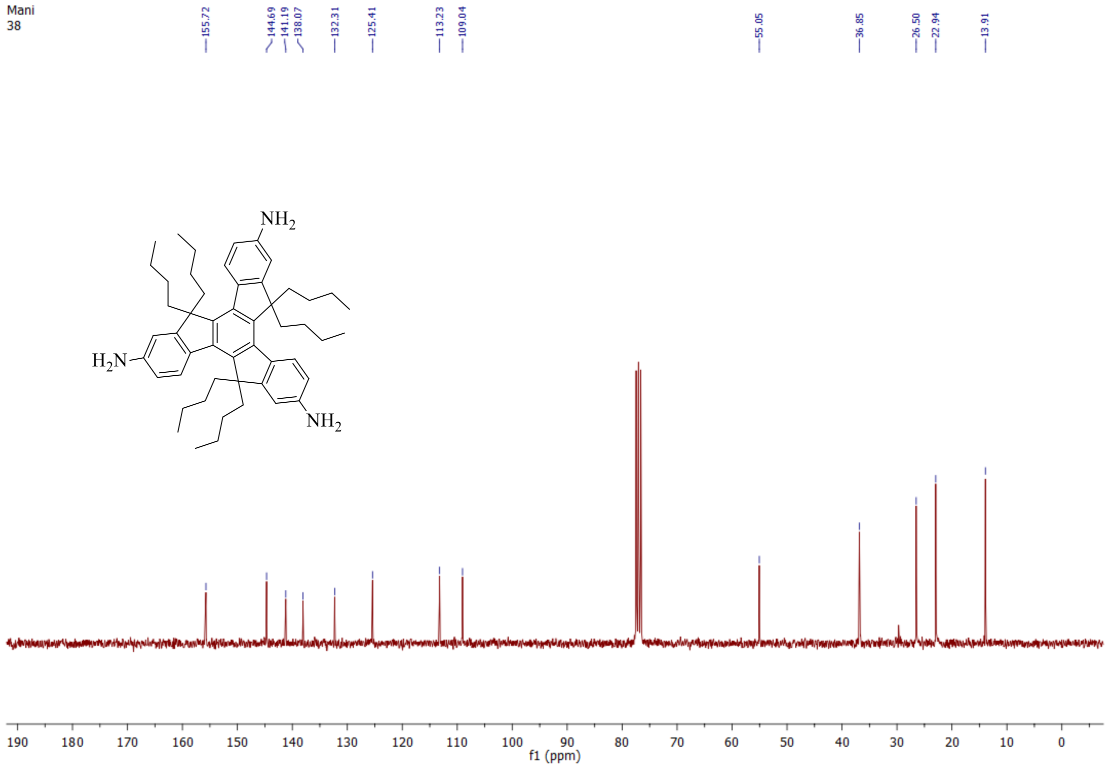

Mass spectra of compound 4

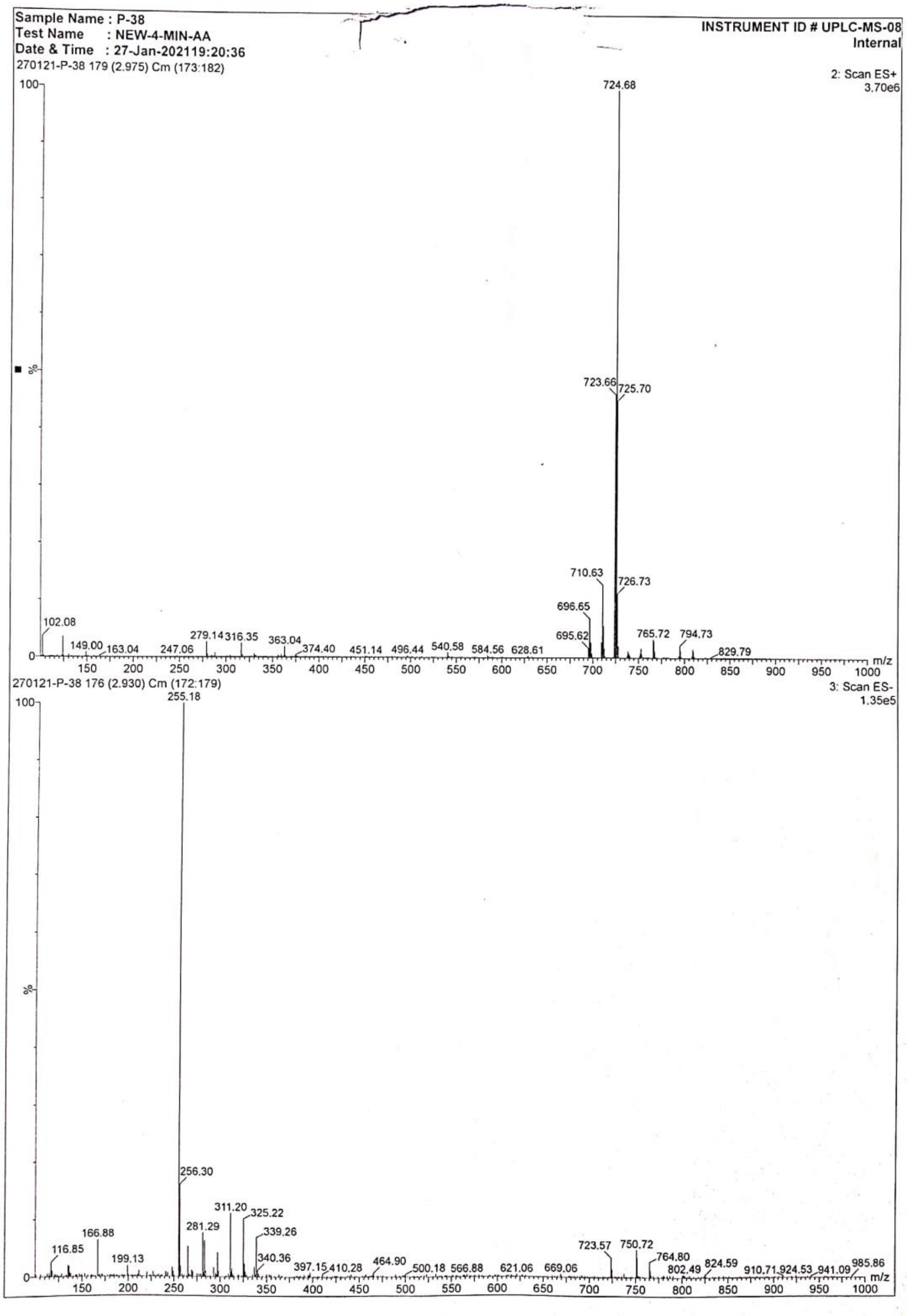

IR spectrum of compound 4

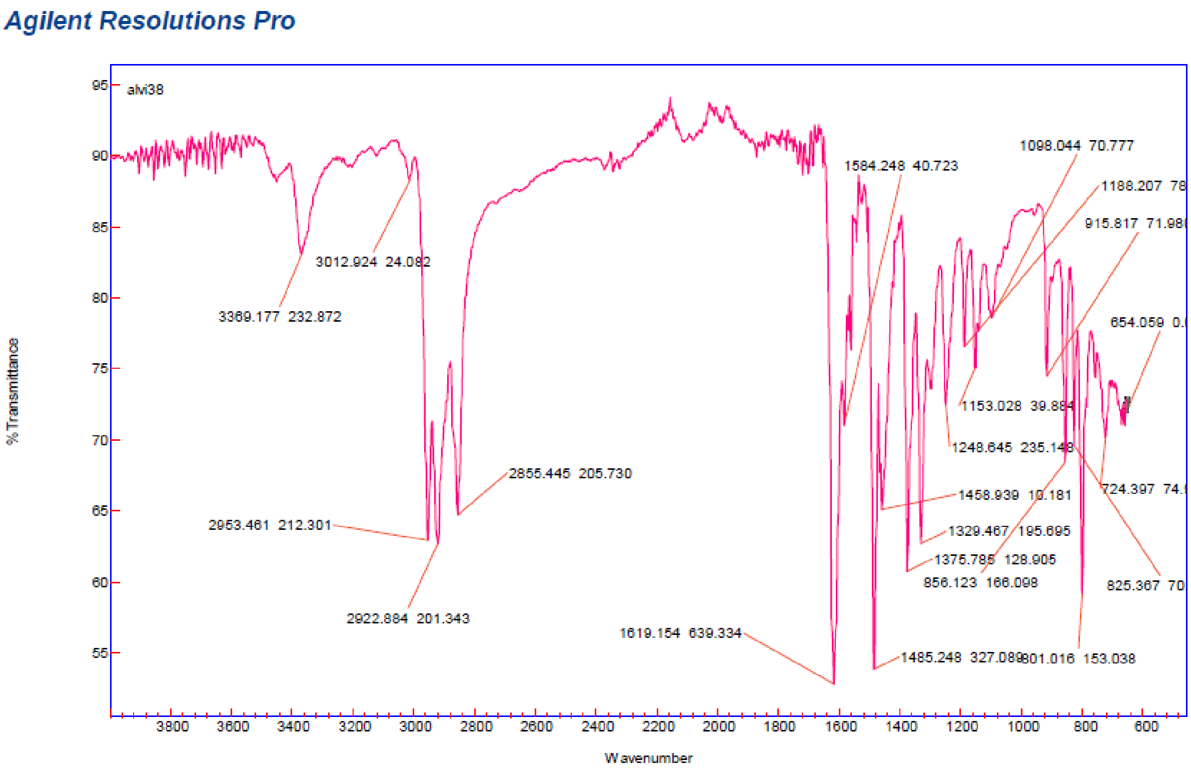

^{1}H NMR spectrum of compound 6

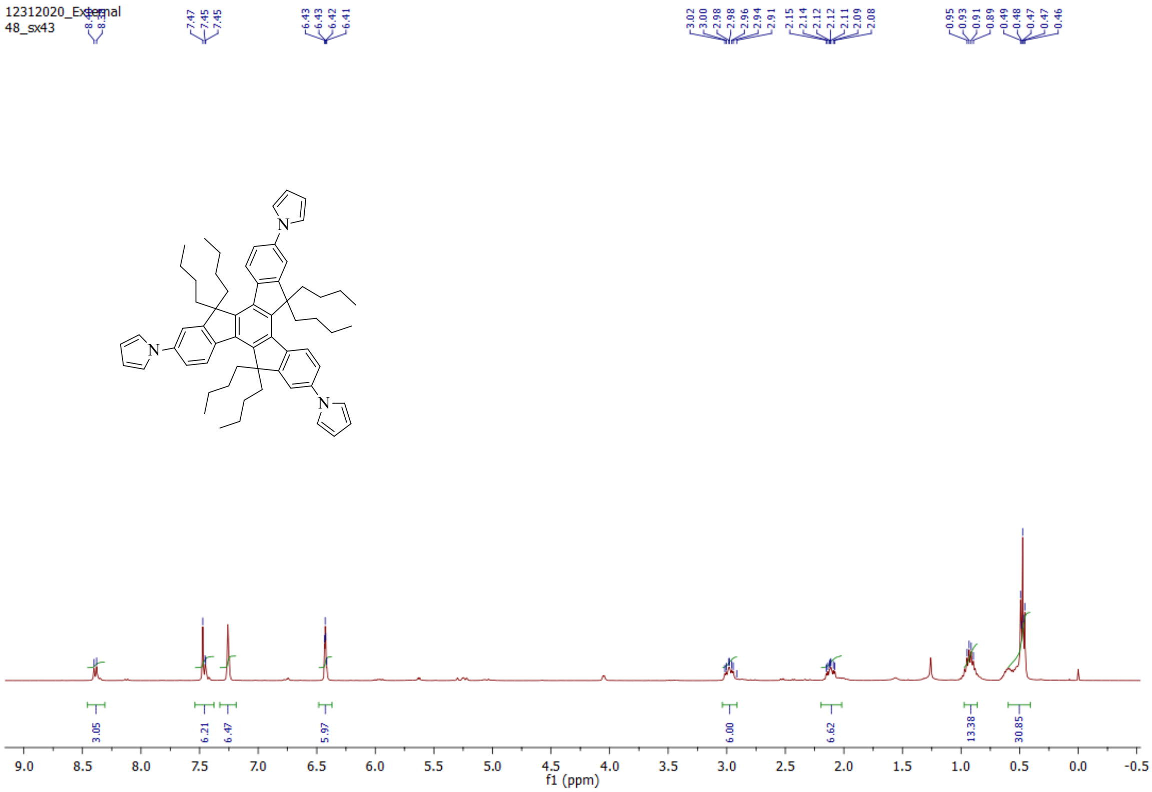

^{13}C NMR spectrum of compound 6

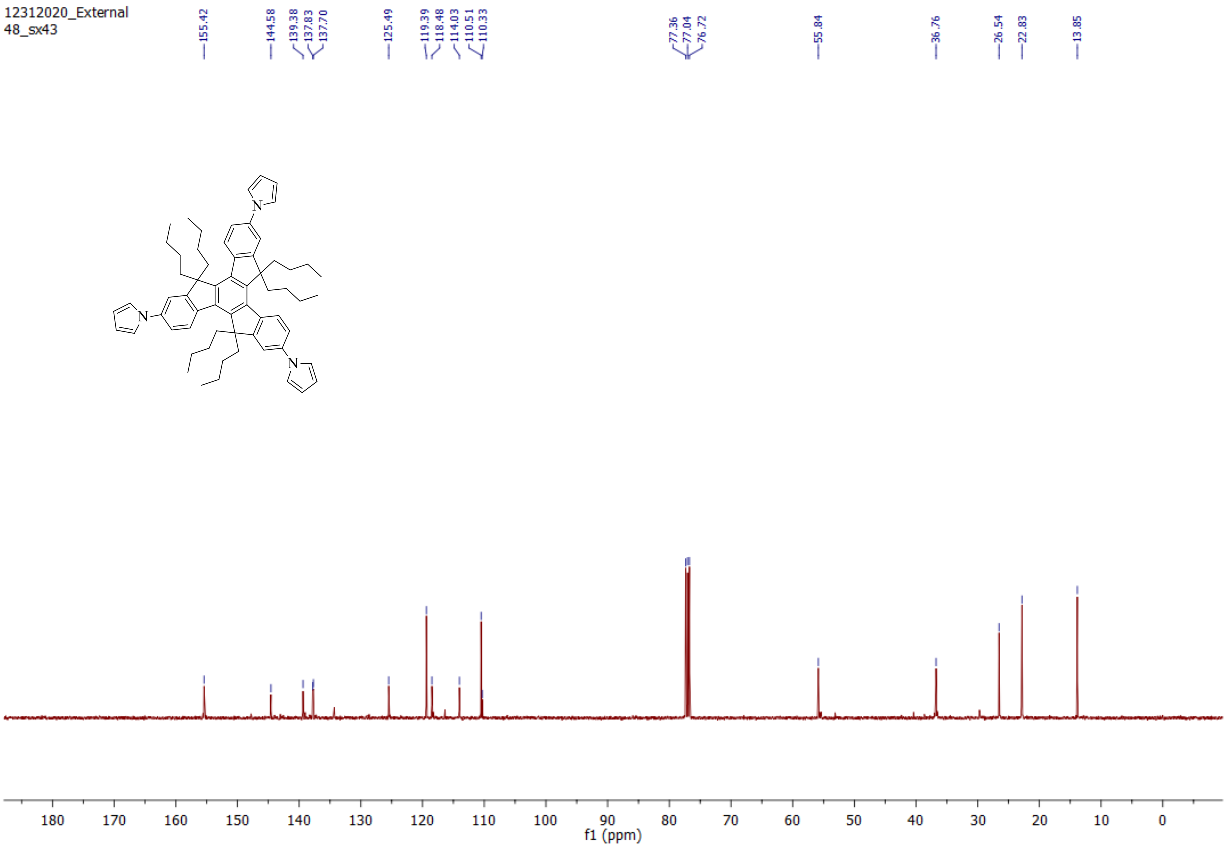

Mass spectra of compound 6

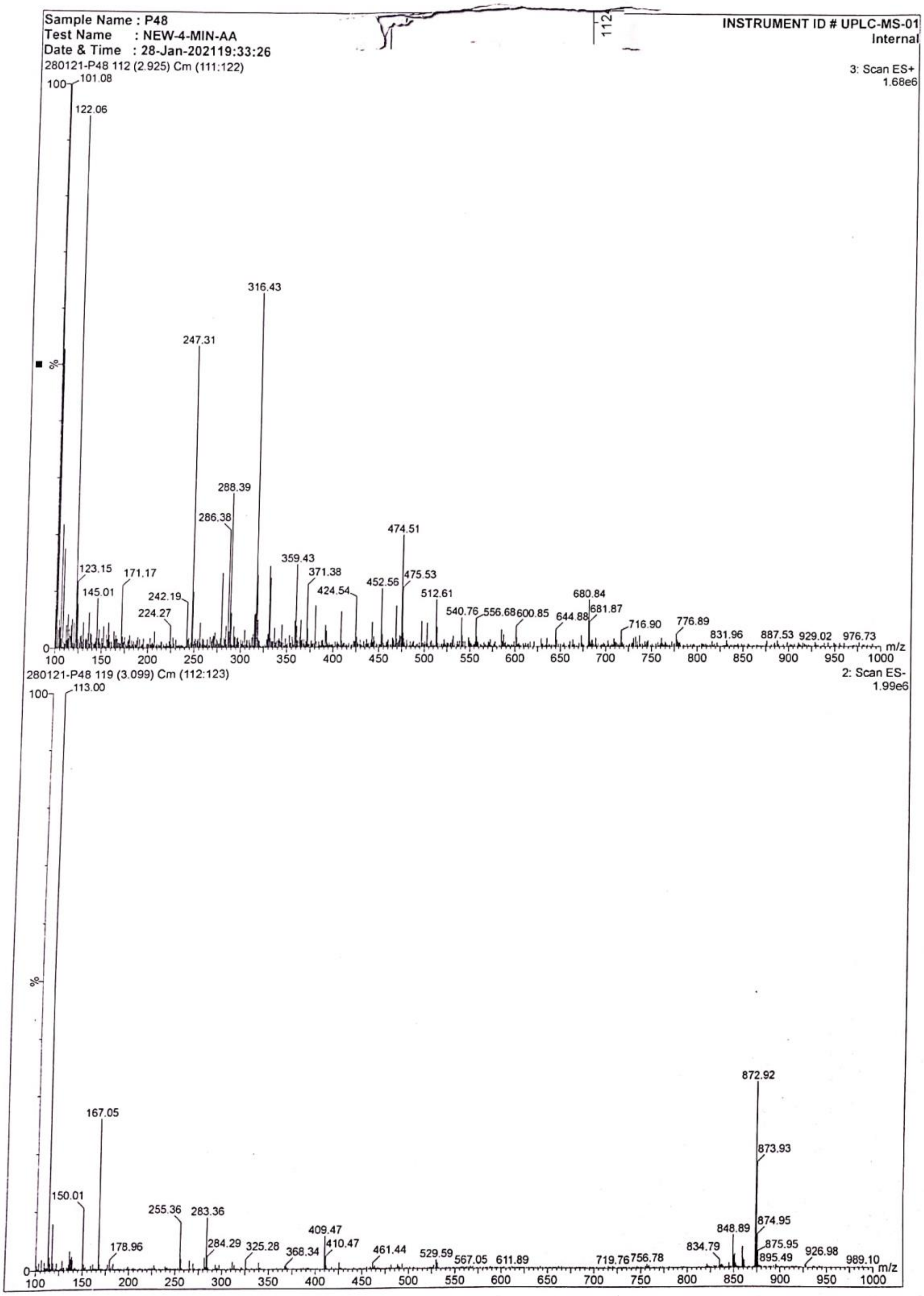

IR spectrum of compound 6

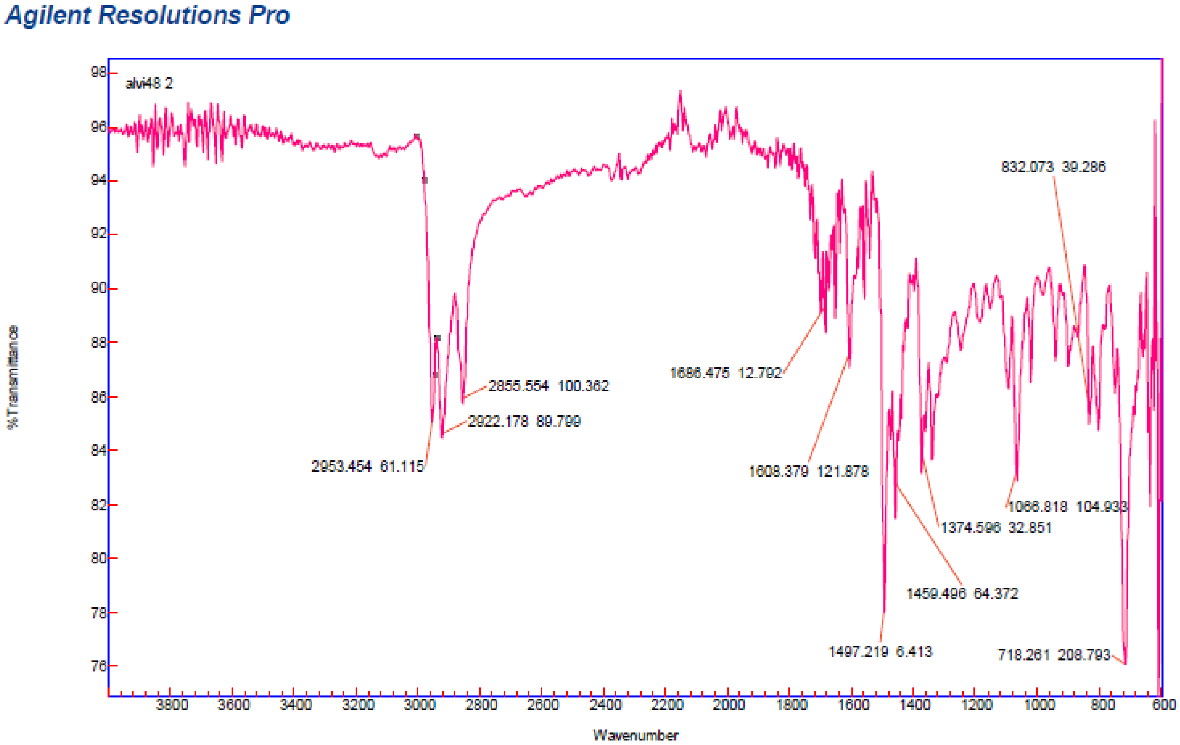

^{1}H NMR spectrum of compound 5

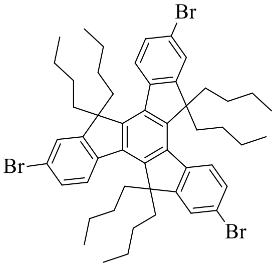

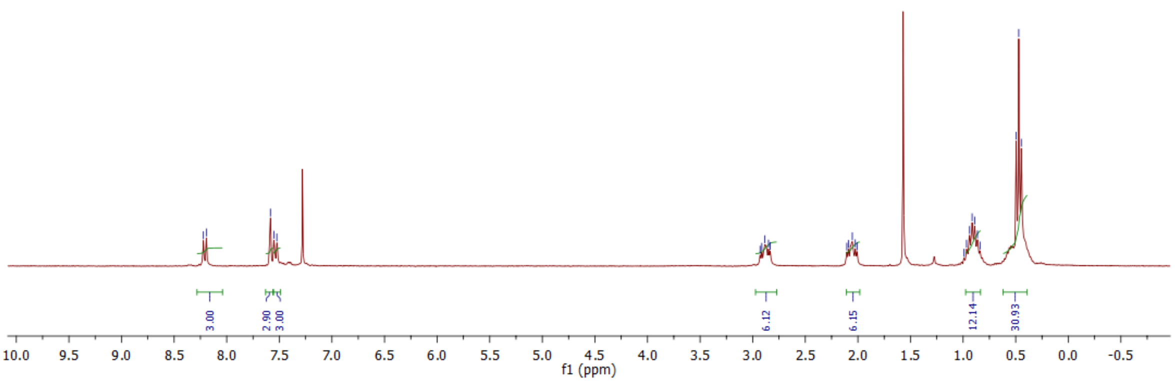

^{1}H NMR spectrum of compound 14

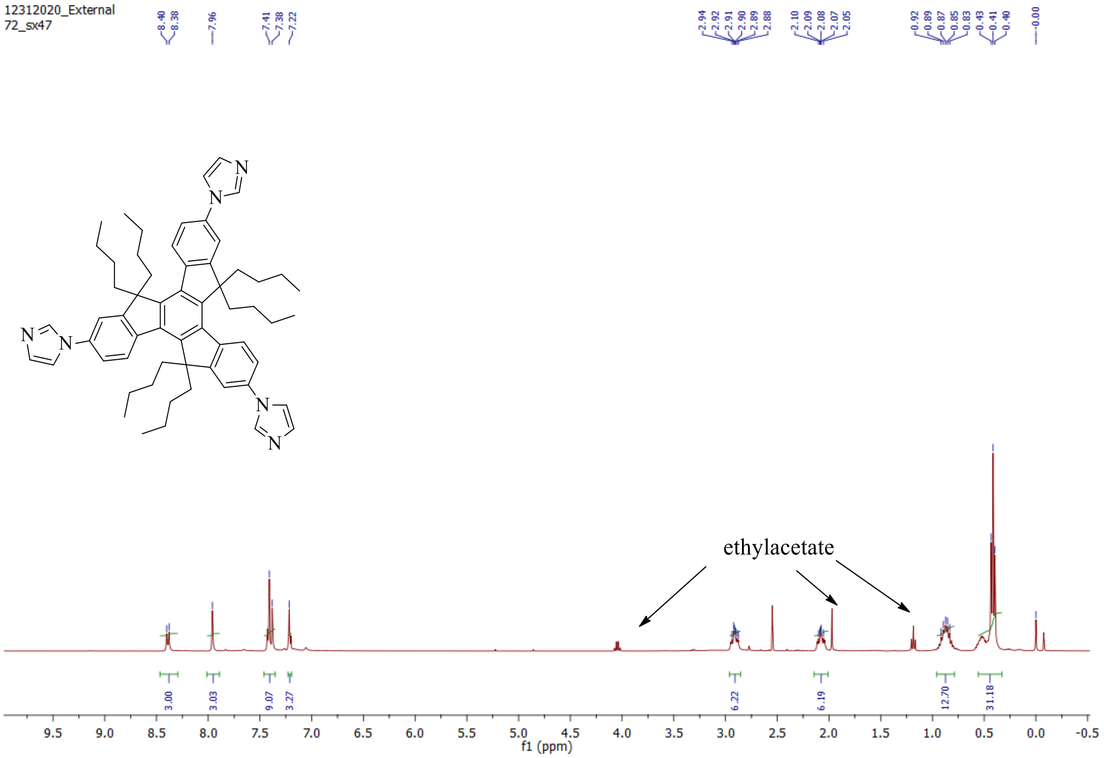

^{13}C NMR spectrum of compound 14

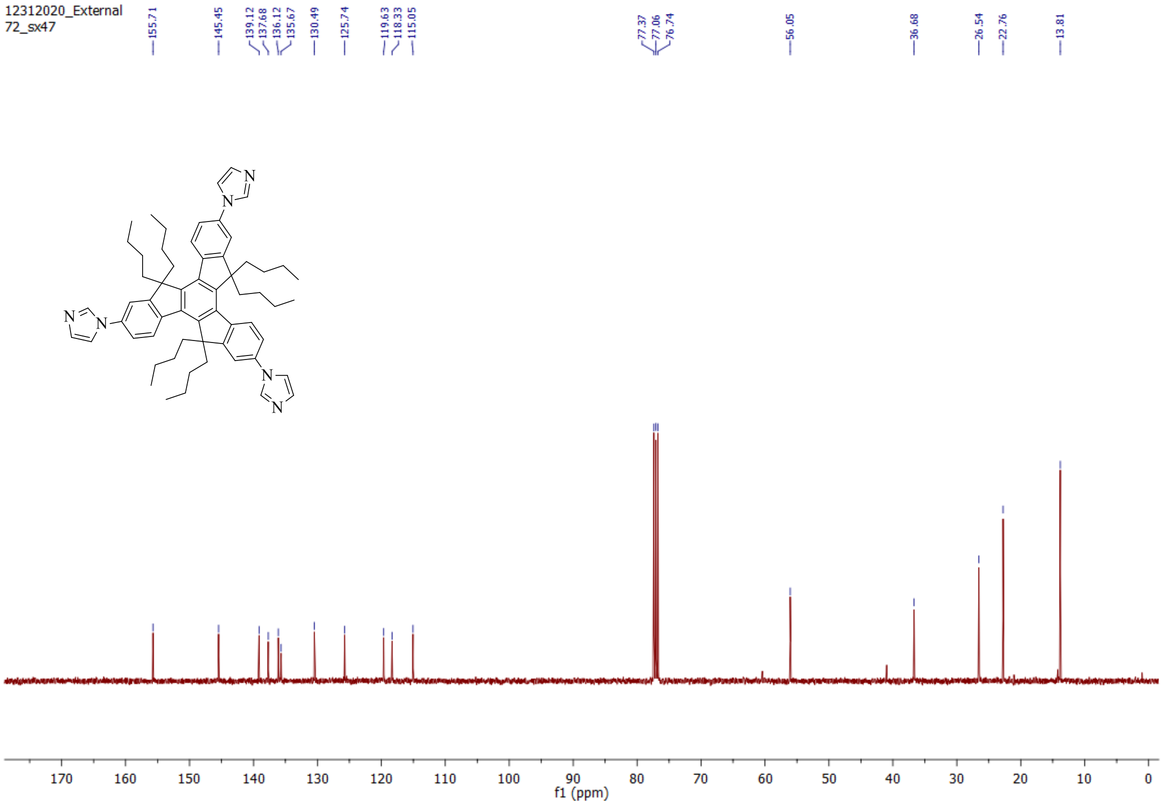

Mass spectra of compound 14

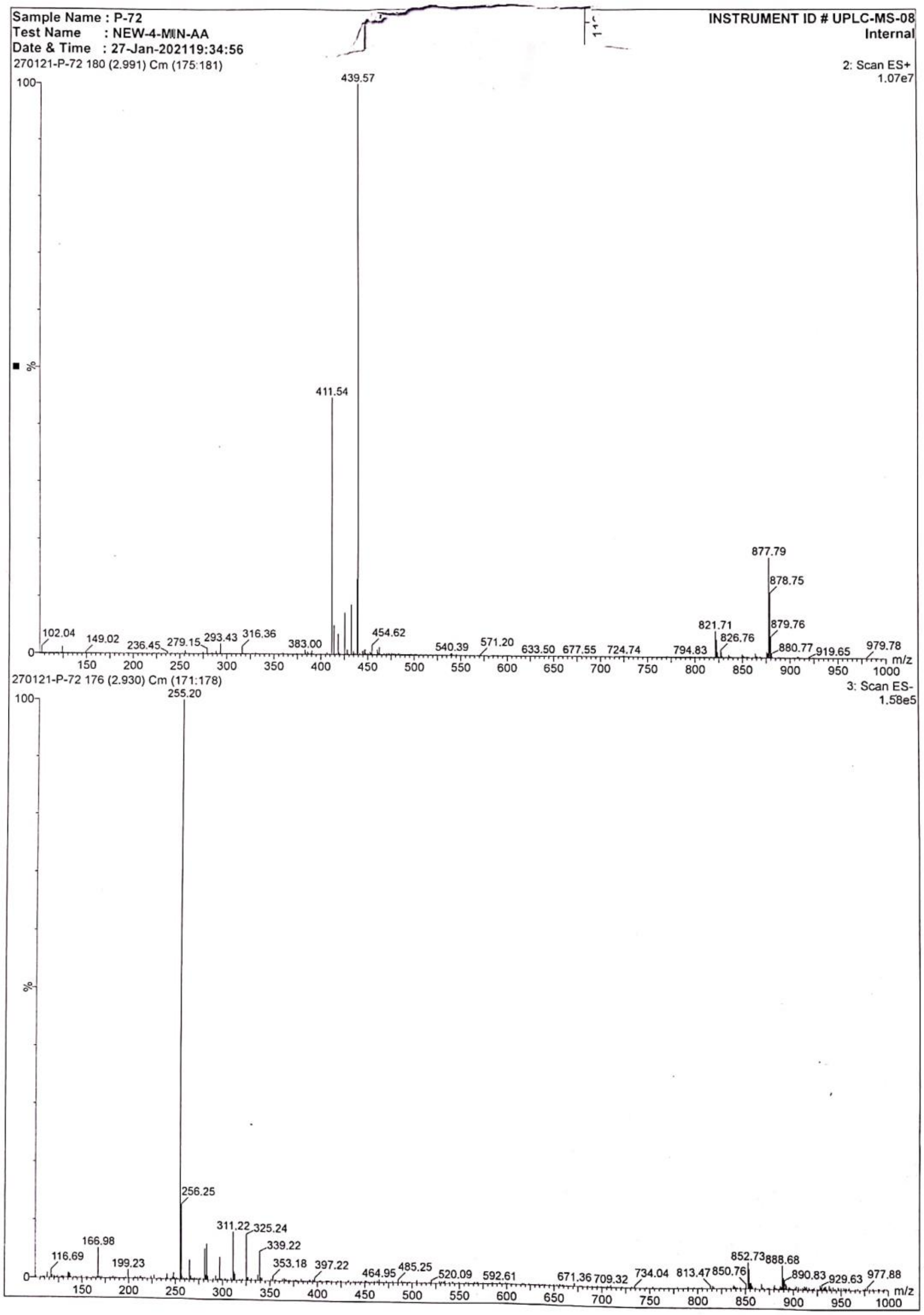

IR spectrum of compound 14

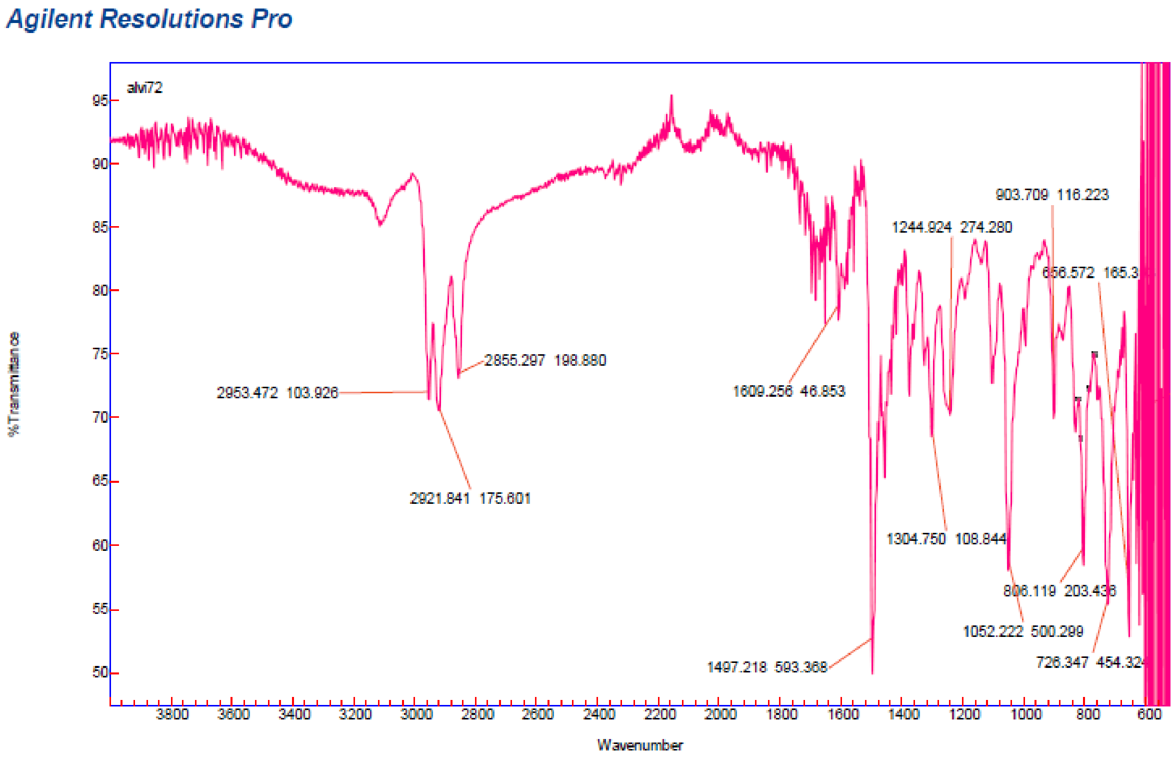

^{1}H NMR spectrum of compound 16

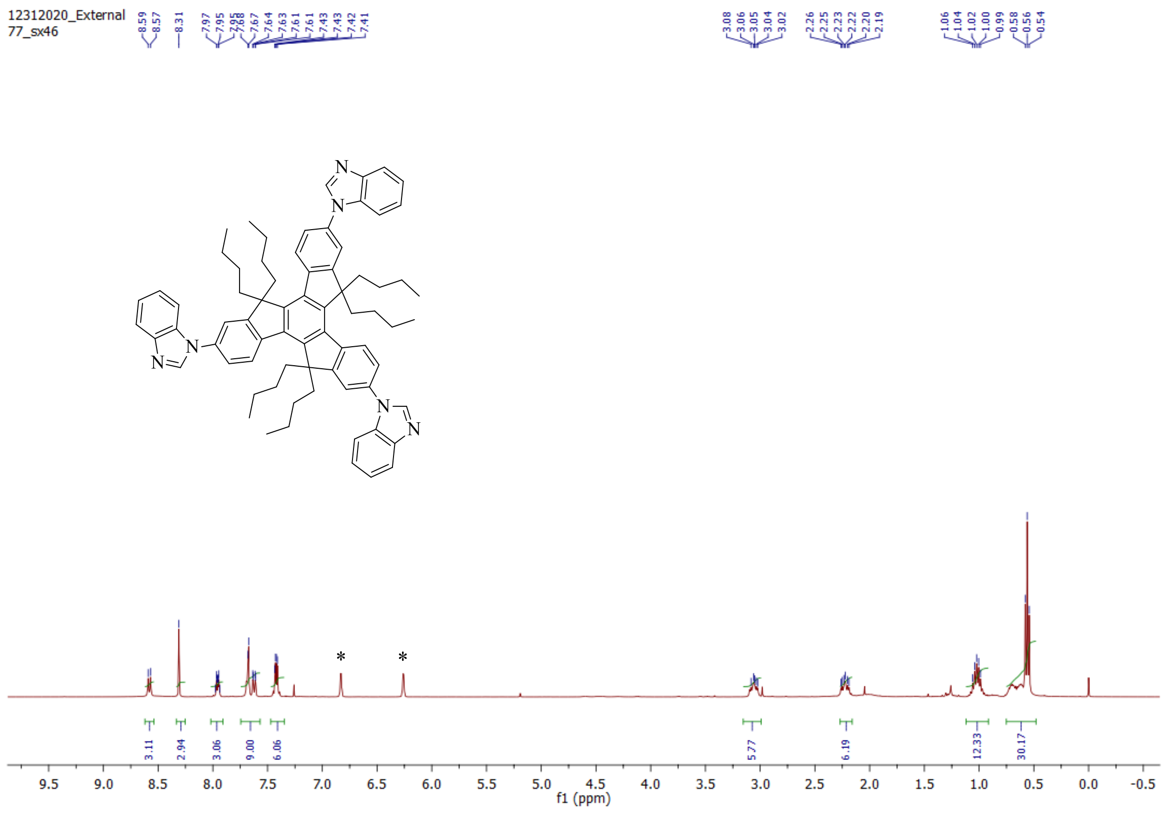

^{13}C NMR spectrum of compound 16

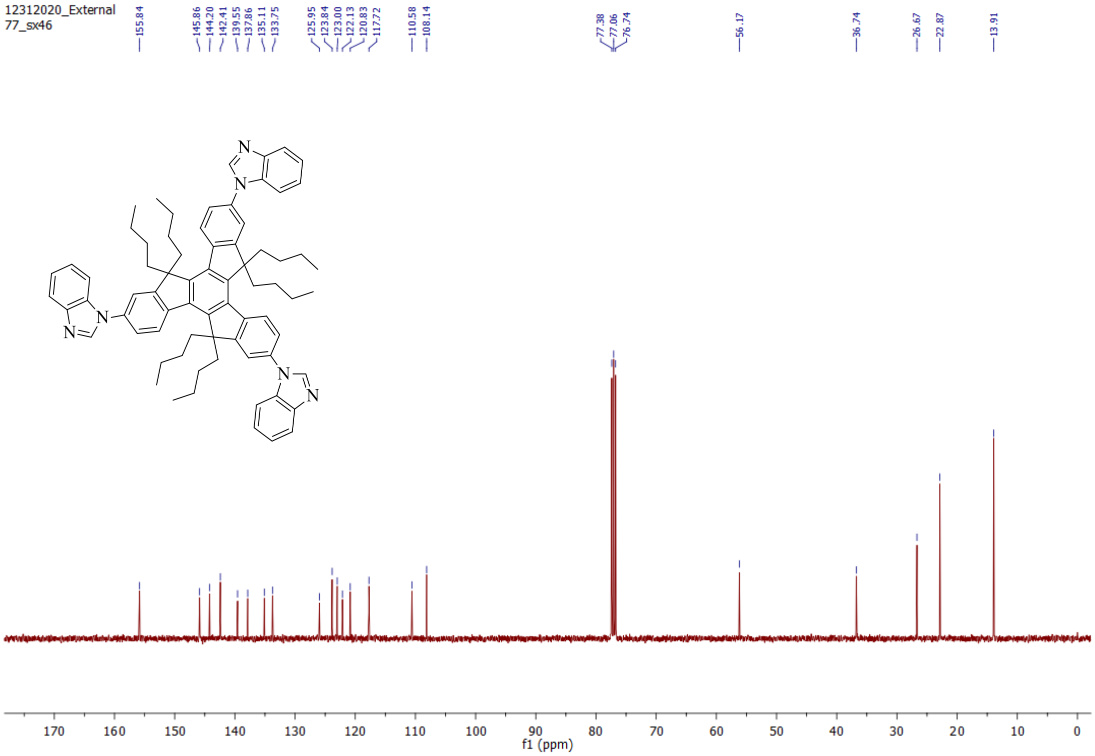

Mass spectra of compound 16

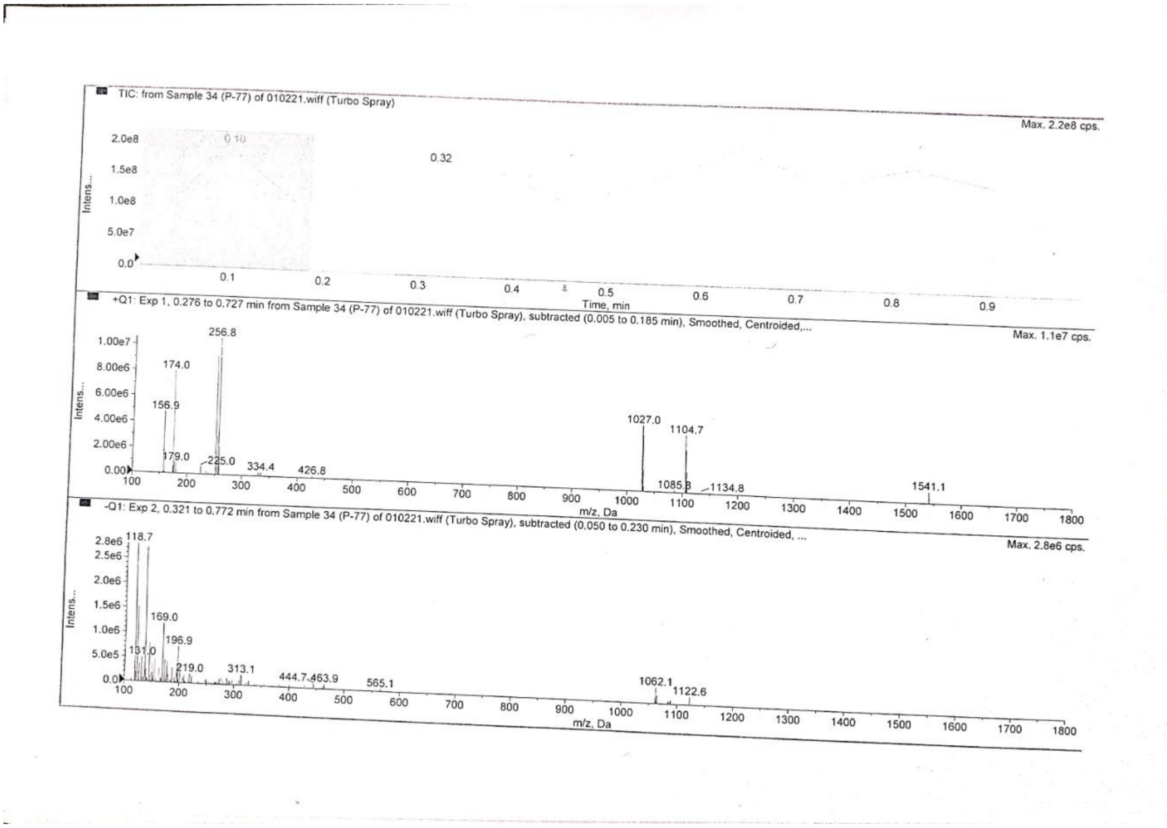

IR spectrum of compound 16

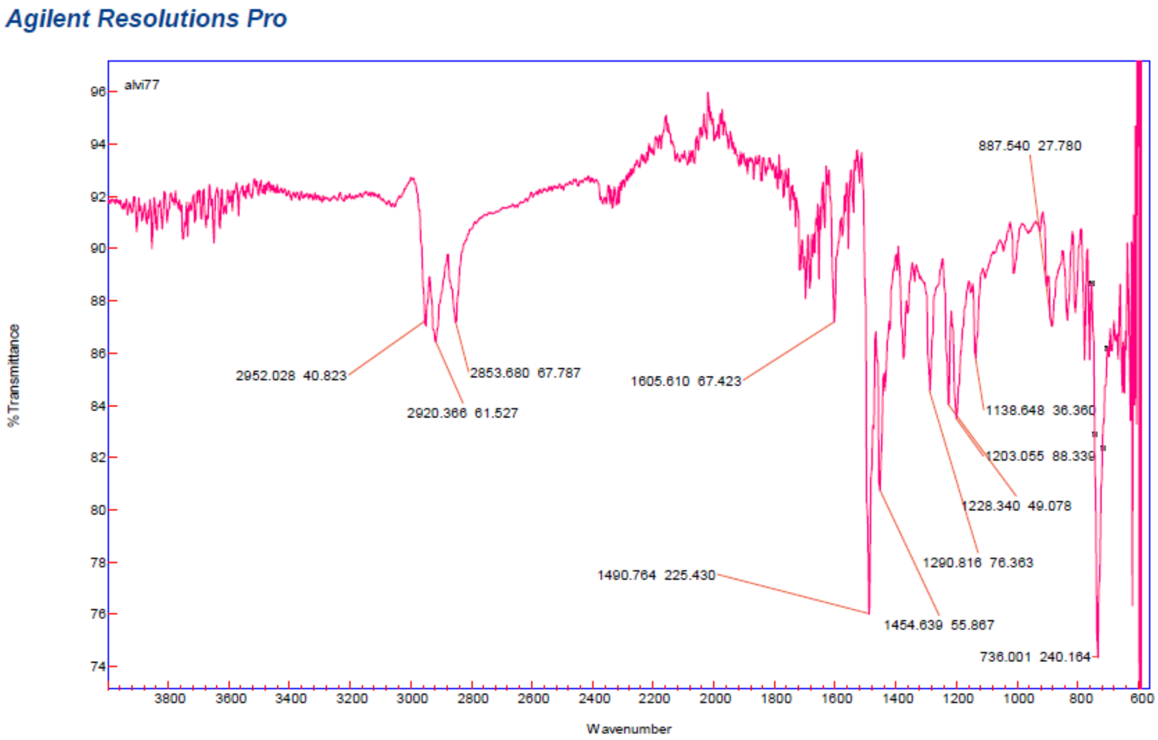

^{1}H NMR spectrum of compound 18

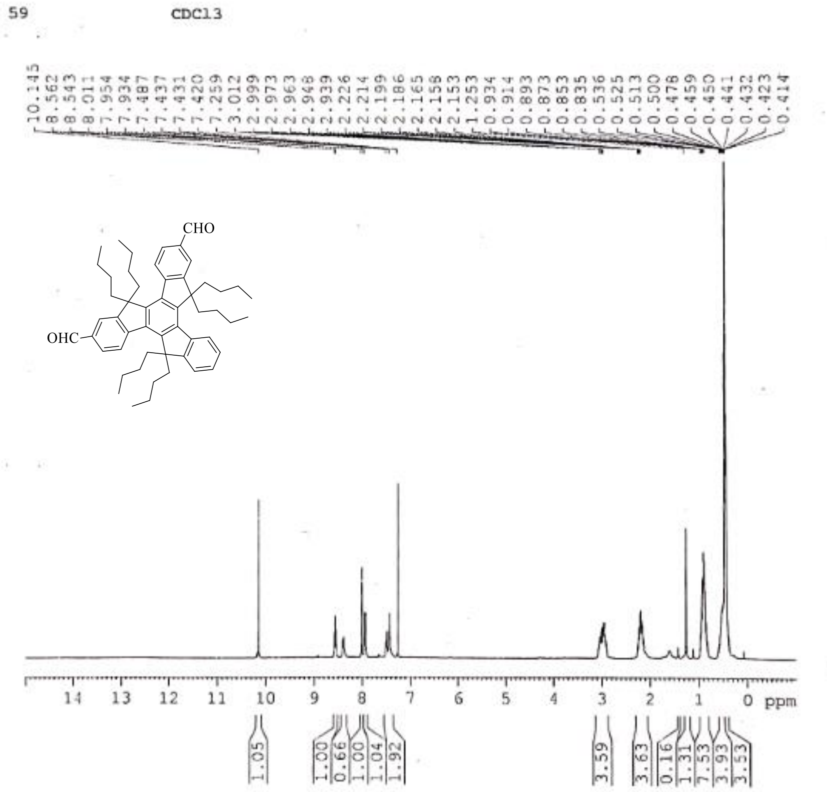

^{1}H NMR spectrum of compound 21

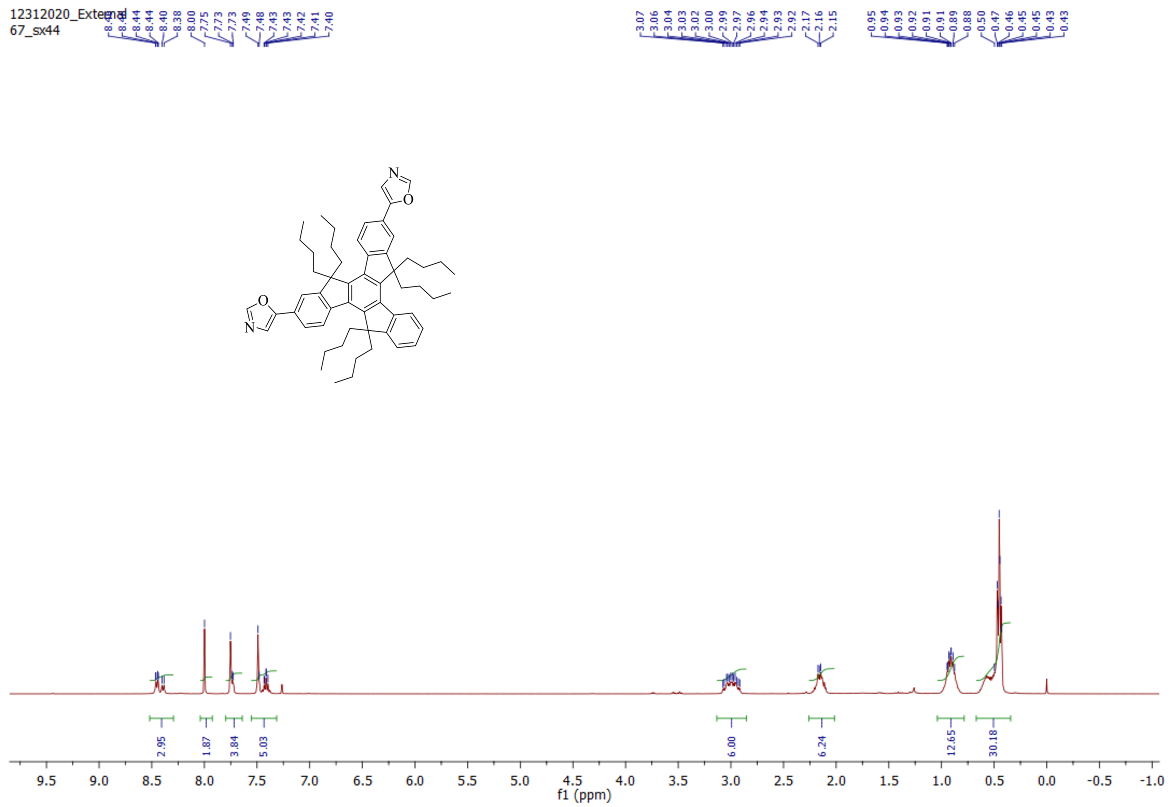

^{13}C NMR spectrum of compound 21

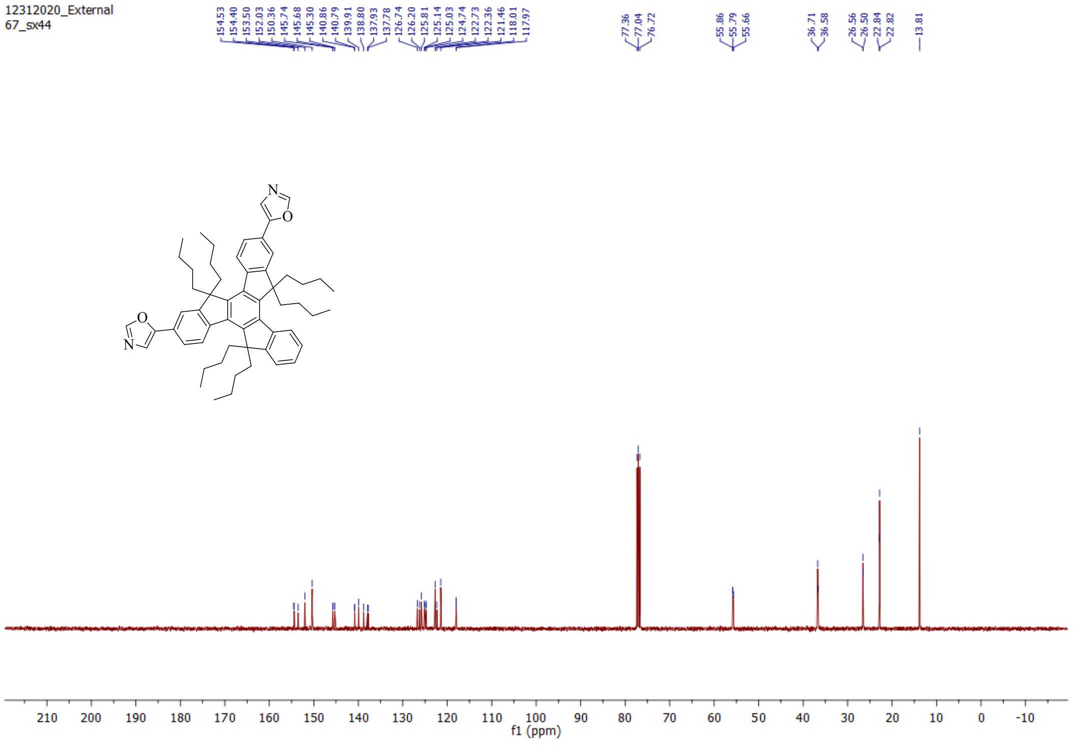

Mass spectra of compound 21

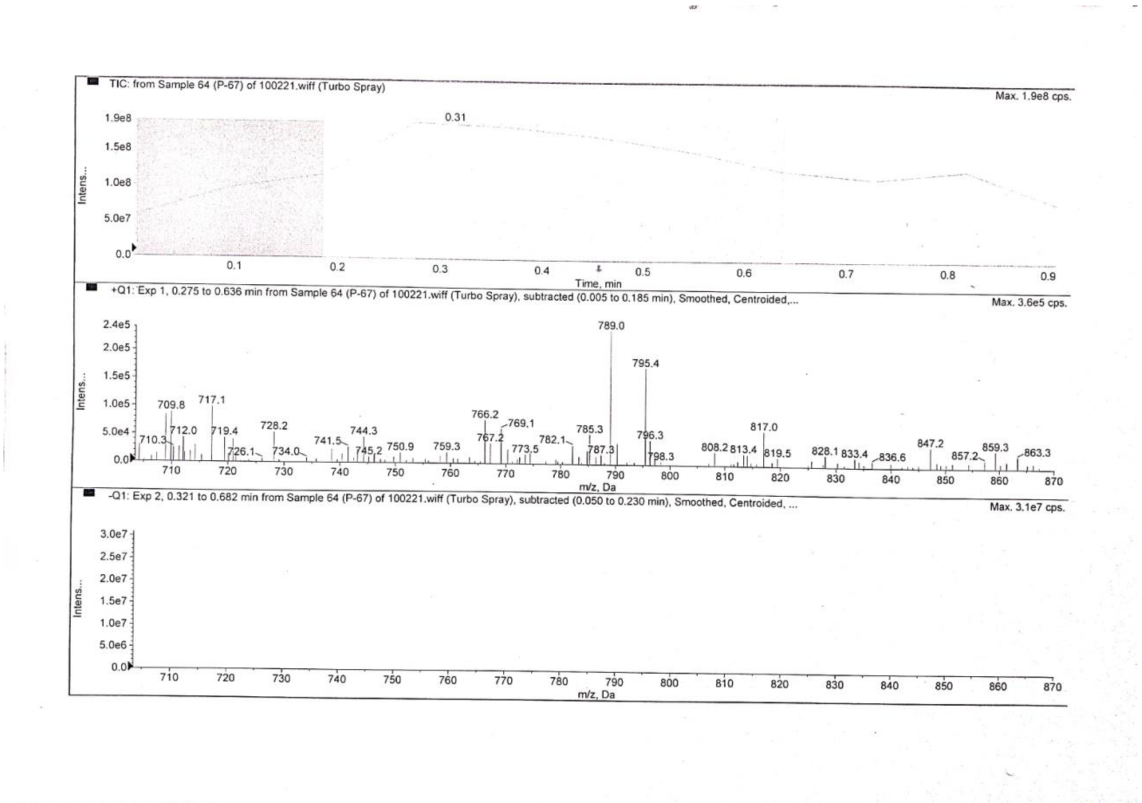

IR spectrum of compound 21

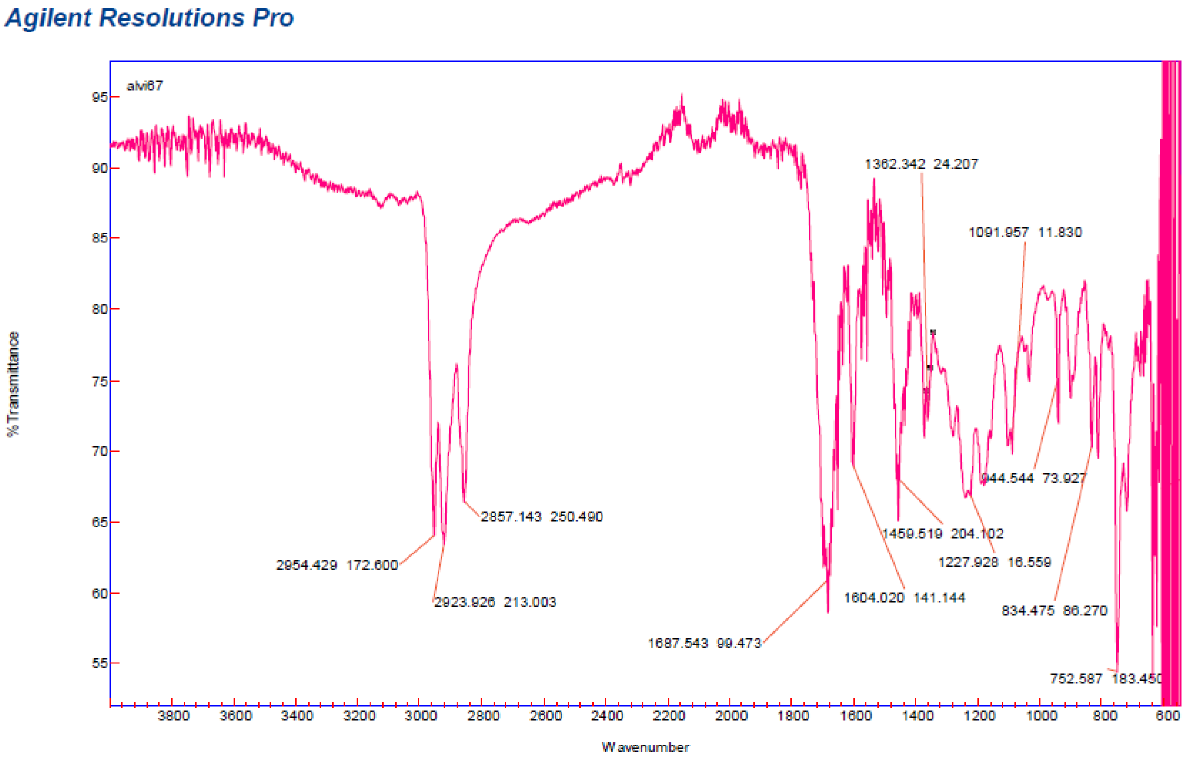

^{1}H NMR spectrum of compound 20

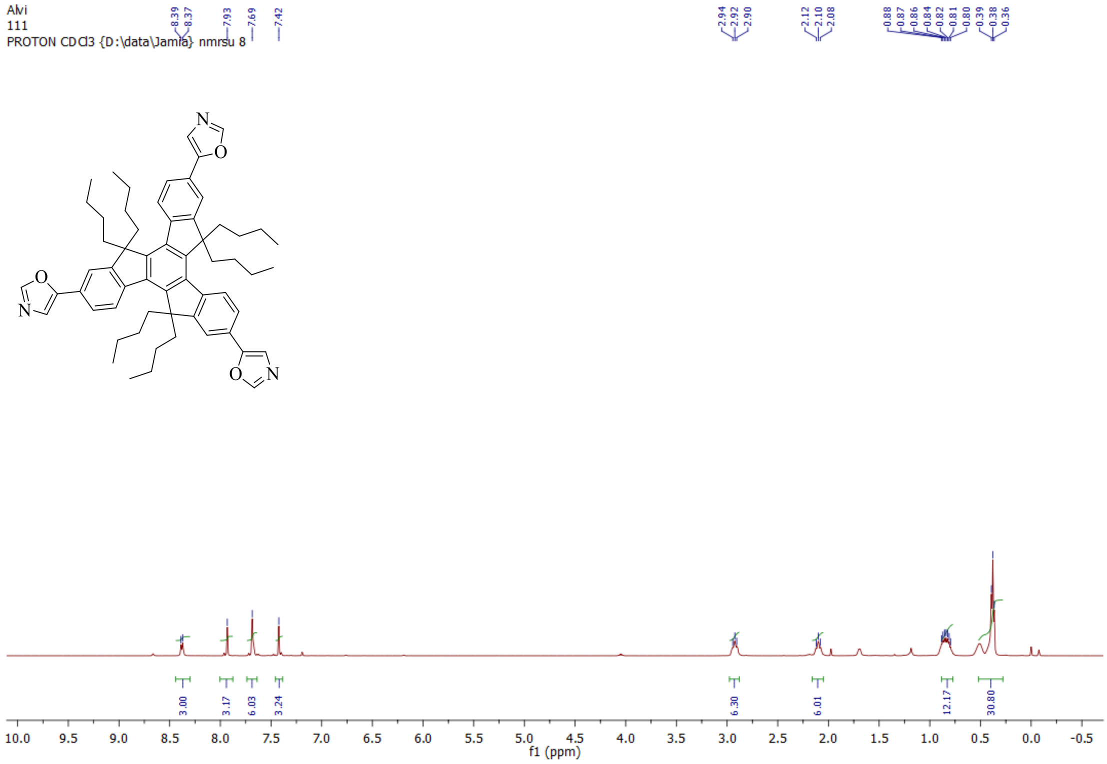

^{13}C NMR spectrum of compound 20

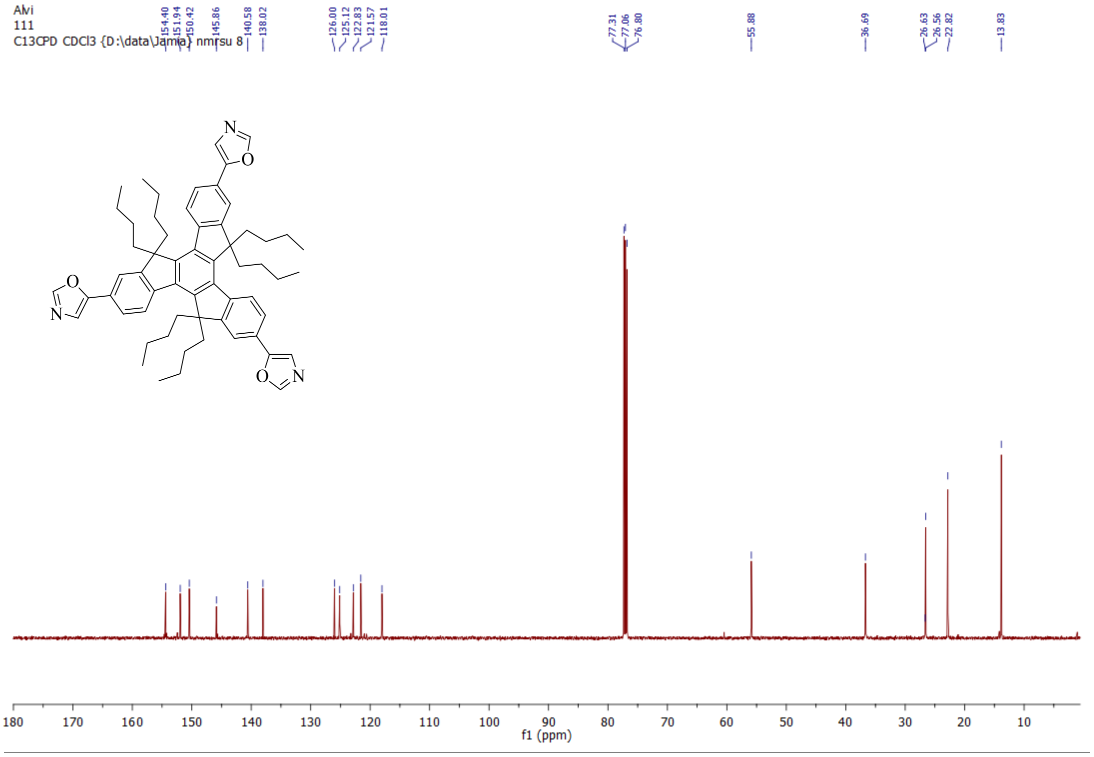

Mass spectra of compound 20

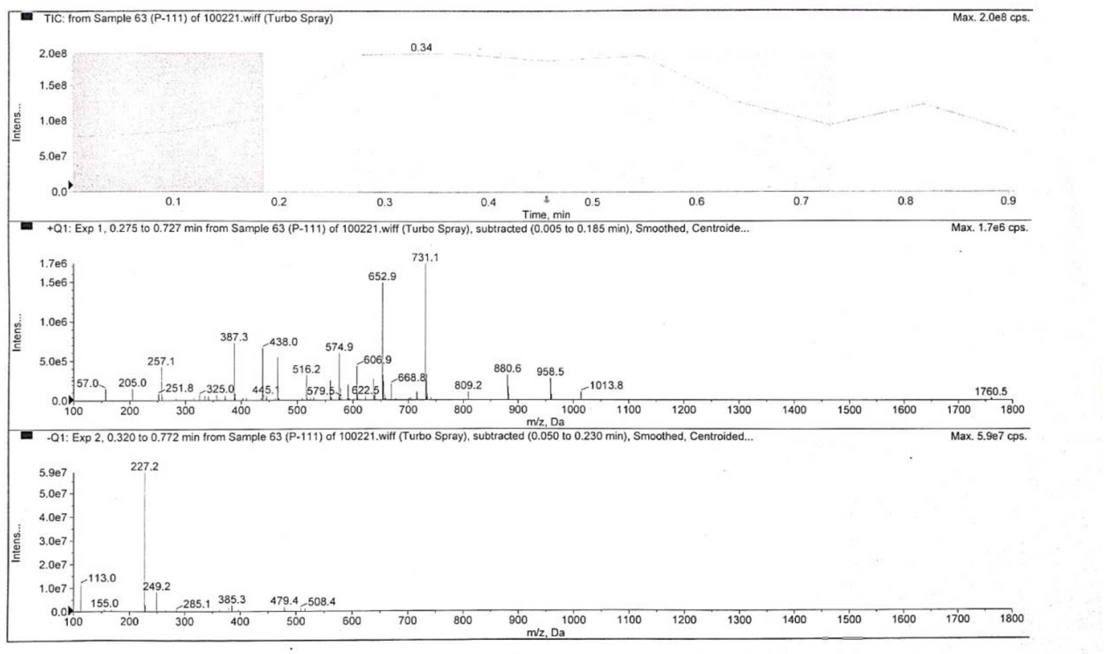

IR spectrum of compound 20

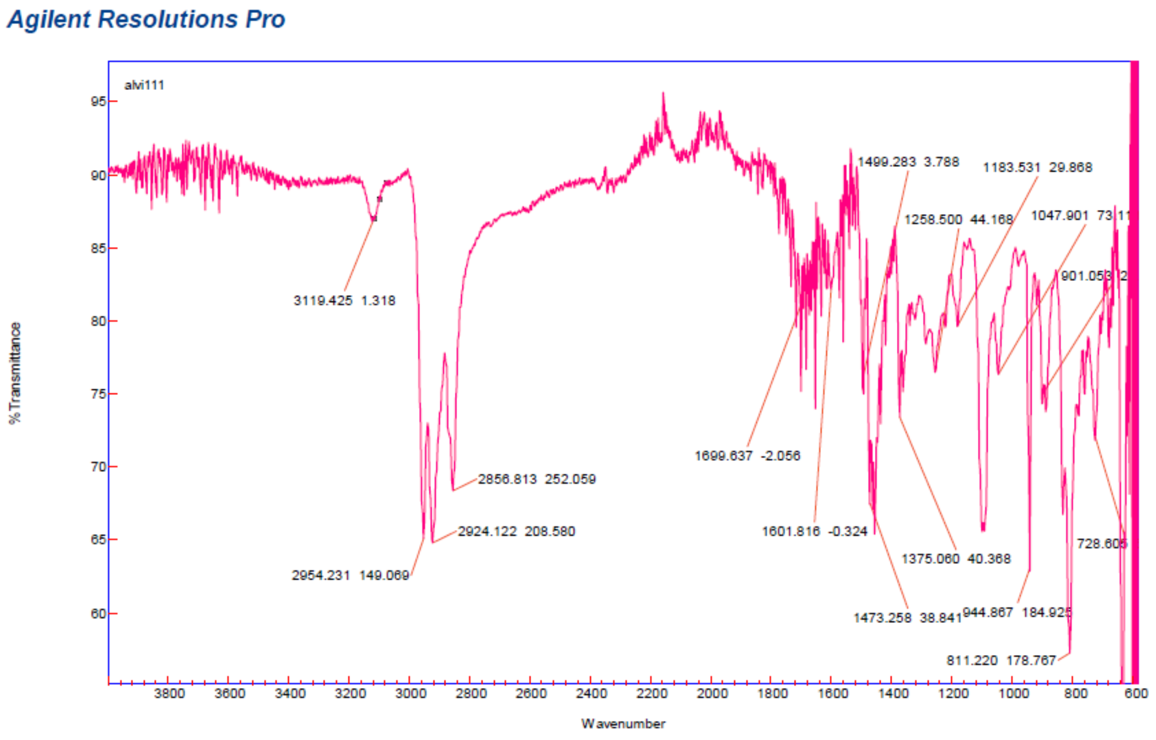

^{1}H NMR spectrum of compound 25

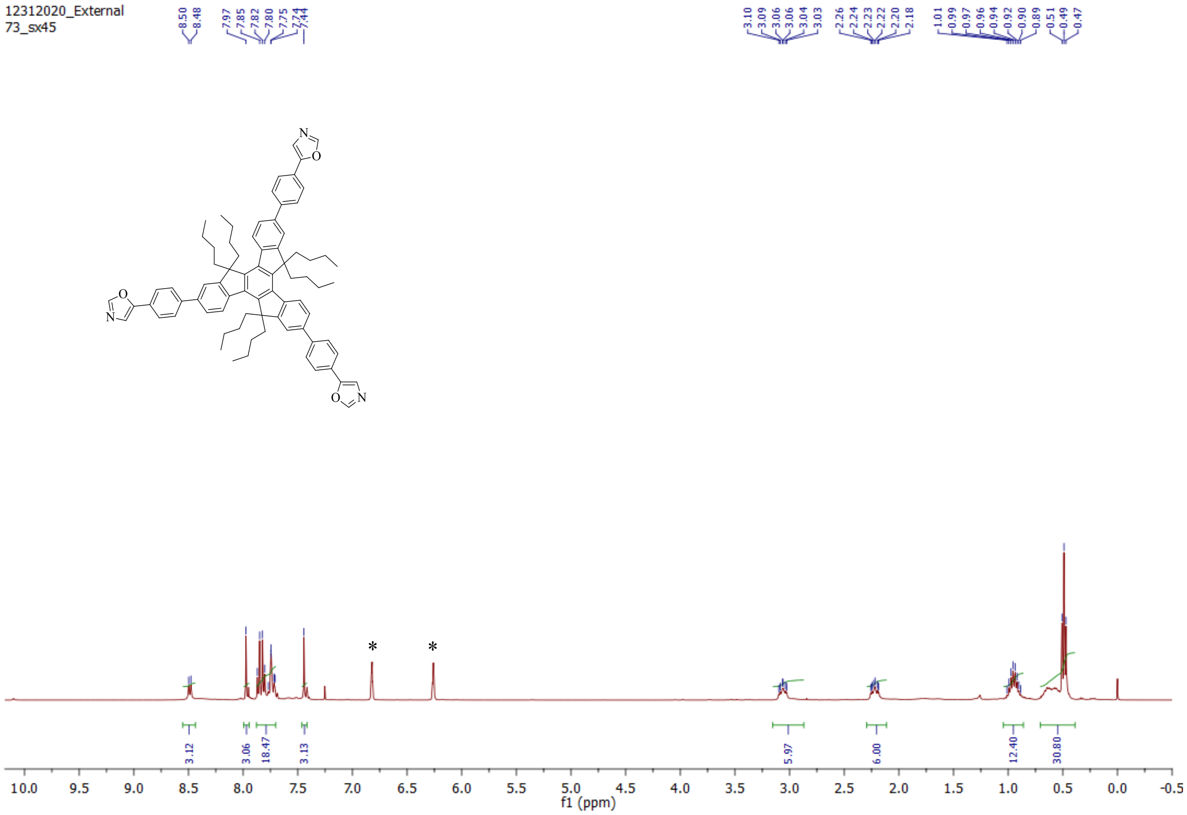

^{13}C NMR spectrum of compound 25

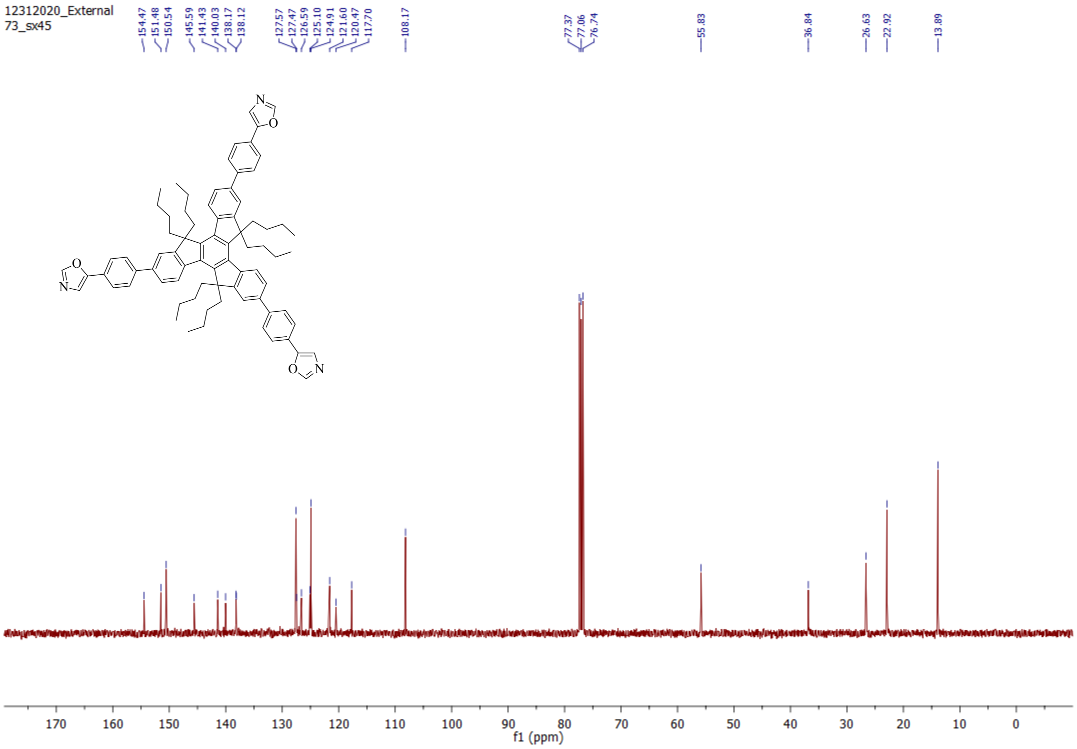

Mass spectra of compound 25

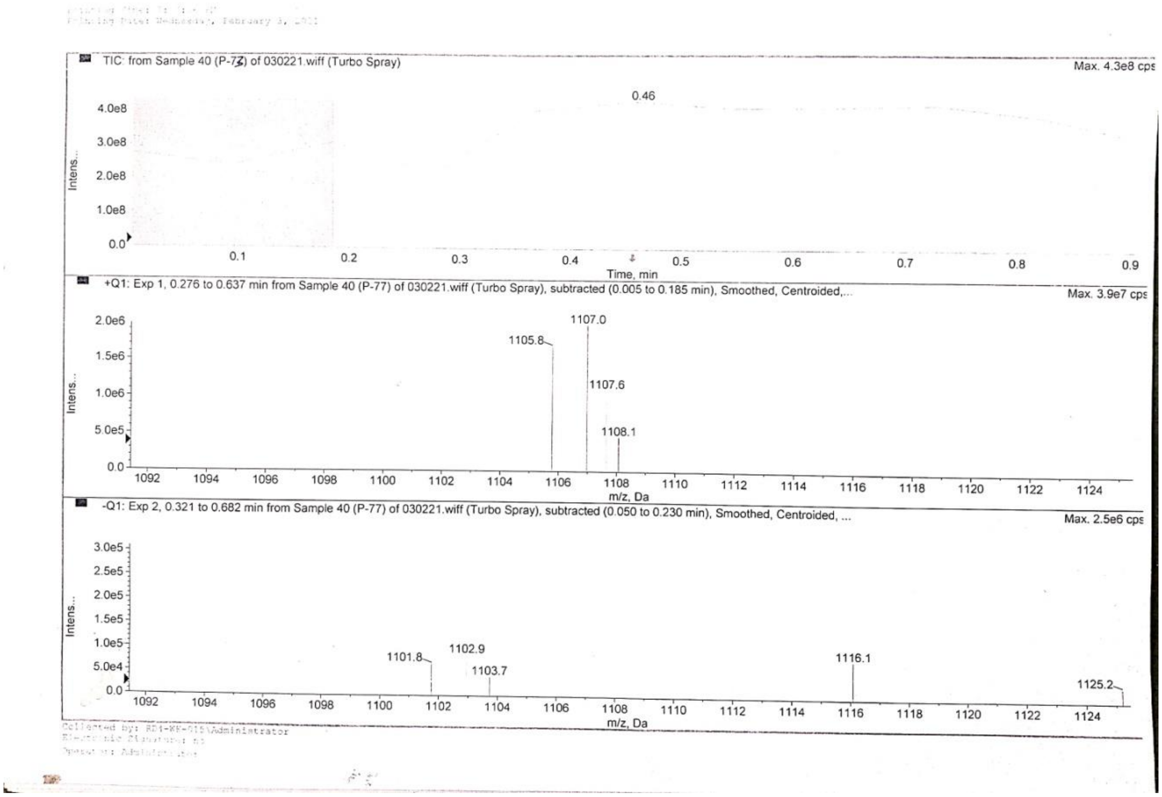

IR spectrum of compound 25

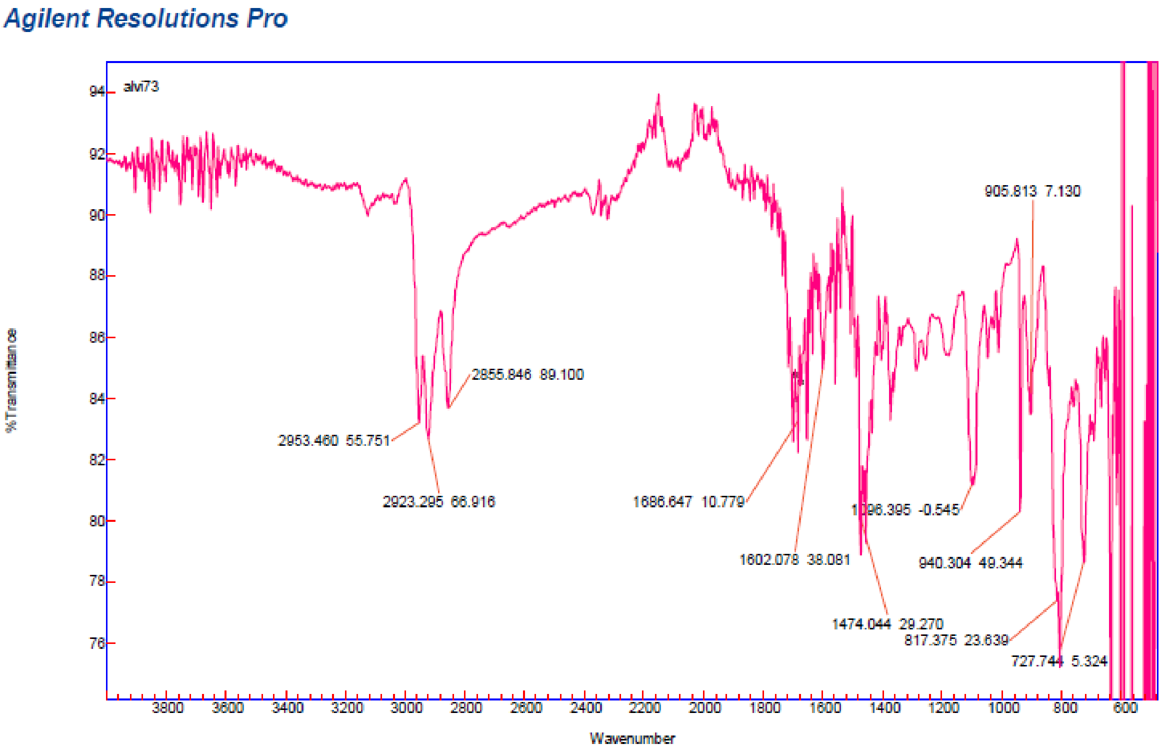

^{1}H-NMR spectrum of compound 22

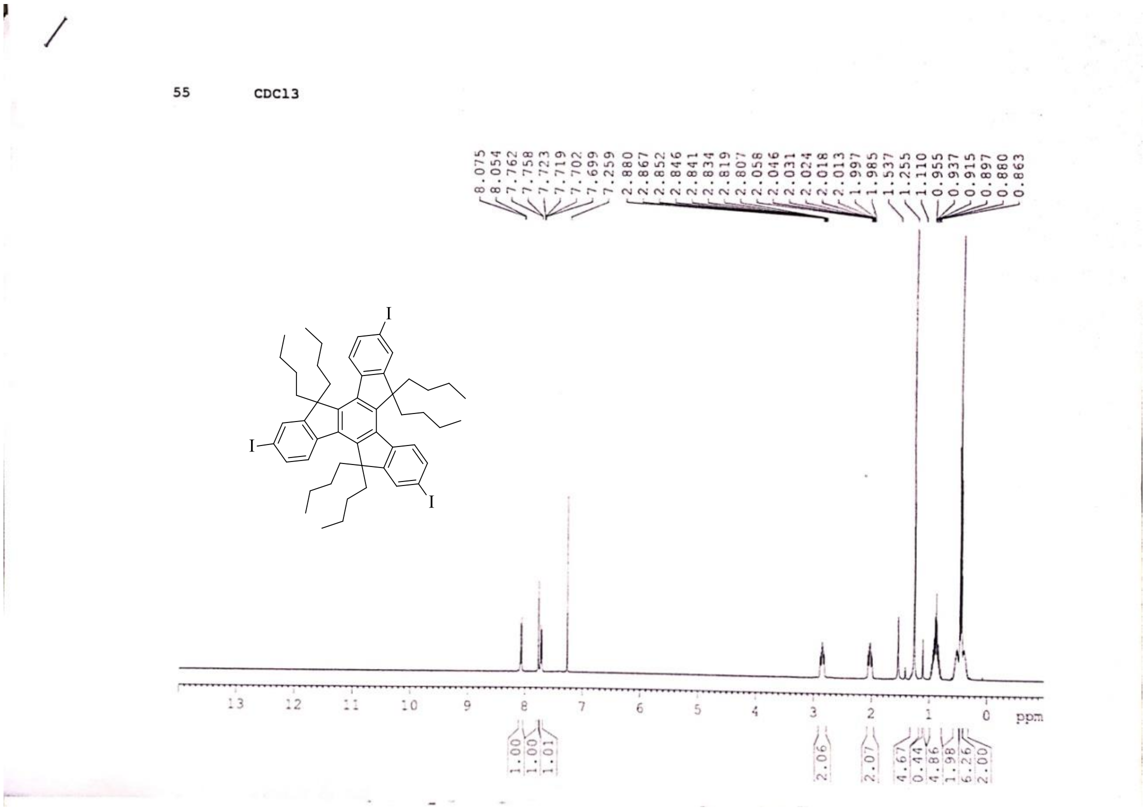

References

1. Langlois, A.; Xu, H.-J.; Brizet, B.; Denat, F.; Barbe, J.-M.; Gros, C. P.; Harvey, P. D. *J. Porphyr. Phthalocyanines* **2014**, *18*, 94–106. doi: 10.1142/S1088424613501150
2. Wang, X.; Wang, Y.; Yang, H.; Fang, H.; Chen, R.; Sun, Y.; Zheng, N.; Tan, K.; Lu, X.; Tian, Z.; Cao, X. *Nat. Commun.* **2016**, *7*, 12469. doi:10.1038/ncomms12469
3. Cao, X.-Y.; Zi, H.; Zhang, W.; Lu, H.; Pei, J. *J. Org. Chem.* **2005**, *70*, 3645–3653. doi:10.1021/jo0480139
